# Predictors of response and rational combinations for the novel MCL‐1 inhibitor MIK665 in acute myeloid leukemia

**DOI:** 10.1002/1878-0261.70130

**Published:** 2025-09-21

**Authors:** Joseph Saad, Rhiannon Newman, Elmira Khabusheva, Sofia Aakko, Eric Durand, Mahesh Tambe, Heikki Kuusanmäki, Alun Parsons, Juho J. Miettinen, Komal Kumar Javarappa, Ezgi June Olgac, Nemo Ikonen, Mika Kontro, Kimmo Porkka, Heiko Maacke, Janghee Woo, Ensar Halilovic, Caroline A. Heckman

**Affiliations:** ^1^ Institute for Molecular Medicine Finland (FIMM), Helsinki Institute of Life Science (HiLIFE), iCAN Digital Precision Cancer Medicine Flagship University of Helsinki Finland; ^2^ Novartis Biomedical Research Basel Switzerland; ^3^ JSS Academy of Higher Education and Research (JSS AHER) University Sophisticated Instrumentation Center (USIC) Mysuru India; ^4^ Department of Hematology Helsinki University Hospital Comprehensive Cancer Center Finland; ^5^ Foundation for the Finnish Cancer Institute Helsinki Finland; ^6^ Winship Cancer Institute of Emory University Atlanta GA USA; ^7^ Novartis Biomedical Research Cambridge MA USA

**Keywords:** acute myeloid leukemia, apoptosis, combination therapies, MCL‐1 inhibition, multidrug resistance‐associated proteins, prognostic biomarkers

## Abstract

Despite promising anti‐leukemic activity of MCL‐1 inhibitors in preclinical studies of acute myeloid leukemia (AML), clinical progress has been hindered by limited knowledge of target patient subgroups. To stratify patients for MCL‐1 inhibitor treatment, we evaluated the sensitivity of 42 primary AML samples to MCL‐1 inhibitor MIK665 (S64315) and analyzed their molecular profiles. We observed that MIK665‐sensitive samples had a more differentiated phenotype, whereas resistant samples displayed higher levels of ABCB1 (MDR1) and the anti‐apoptotic protein BCL‐XL. Moreover, *ABCB1* expression had good predictive performance in identifying resistant samples. To induce sensitivity, we treated MIK665‐resistant samples with ABCB1 inhibitors elacridar or tariquidar, BCL‐XL inhibitor A1331852, or BCL‐2 inhibitor venetoclax in combination with MIK665. The combination of MIK665 with each of elacridar, tariquidar, or venetoclax effectively eliminated AML blasts compared to the agents alone, while the combination with A1331852 showed limited efficacy for this patient subgroup. Additionally, the combination of MIK665 with venetoclax restored sensitivity in samples with primary venetoclax resistance. Overall, this study indicates that elevated *ABCB1* expression is a potentially targetable resistance mechanism in the context of MIK665 resistance, and that a combination of MIK665 with venetoclax may be effective for overcoming resistance to either MCL‐1 or BCL‐2 inhibition.

## Graphical abstract

This study characterizes the responses of primary acute myeloid leukemia (AML) patient samples to the MCL‐1 inhibitor MIK665. The results revealed that monocytic differentiation is associated with MIK665 sensitivity. Conversely, elevated *ABCB1* expression is a potential biomarker of resistance to the treatment, which can be overcome by the combination of MIK665 with ABCB1 inhibitors or with the BCL‐2 inhibitor venetoclax.

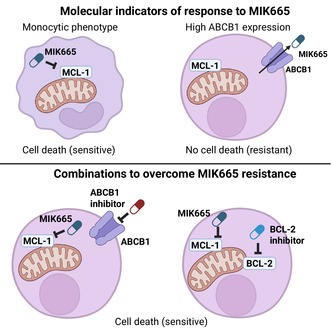

**Abbreviations**
AML: acute myeloid leukemia
AUC: area under the curve
BM: bone marrow
CM: conditioned media
CPM: counts per million
DSS: drug sensitivity score
ELN: European LeukemiaNet
FAB: French American British
FBS: fetal bovine serum
FC: fold change
FDR: false discovery rate
MDR1: multidrug resistance protein 1
MNC: mononuclear cell
NA: not available
P‐gp: P‐Glycoprotein
ROC: receiver operating characteristics
TMM: trimmed mean of *M* values
ZIP: zero interaction potency

## 1 Introduction

The BCL‐2 inhibitor venetoclax is a recently approved targeted therapy for acute myeloid leukemia (AML) in combination with azacitidine or low‐dose cytarabine for newly diagnosed patients unfit for intensive chemotherapy. These treatments have significantly improved the prognosis of this previously difficult‐to‐treat subgroup of patients [1, 2, 3, 4]. Apoptosis is a tightly regulated molecular process essential for the maintenance of cellular homeostasis, the deregulation of which is a fundamental hallmark of tumorigenesis [5]. The dependency of AML blasts on different anti‐apoptotic BCL‐2 family members, such as BCL‐2, MCL‐1, and BCL‐XL, to escape apoptosis marks these proteins as ideal targets for therapeutic inhibition [6]. Despite significant improvements in overall survival and remission rates, venetoclax‐based therapies are challenged by both primary and acquired resistance leading to relapse [7]. This raises the need for novel, rational drug combinations capable of attaining deeper and prolonged responses.

In AML, elevated MCL‐1 expression plays a central role in escape from apoptosis and has been associated with poor patient prognosis [8, 9]. As such, inhibitors targeting MCL‐1 have been evaluated in preclinical studies alone and in combination with BCL‐2 inhibitors, resulting in encouraging findings [10, 11, 12, 13, 14, 15]. These results have supported the clinical investigation as evaluate of several MCL‐1 inhibitors as monotherapies or in combination with BCL‐2 inhibitors and hypomethylating agents [16, 17, 18, 19, 20]. However, the progression of MCL‐1 inhibitors towards clinical approval has been hindered by dose‐limiting cardiotoxicity and limited knowledge of biomarkers of response [15, 16]. Therefore, the identification of patient subgroups likely to benefit from MCL‐1 inhibition, as well as suitable drug combinations, could improve efficacy and accelerate the development of this drug class.

In this study, we evaluated the activity of the MCL‐1 inhibitor MIK665 in AML preclinical models. To identify indicators of response, we assessed the *ex vivo* sensitivity of 42 AML samples to MIK665 and compared the transcriptional and protein expression profiles of MIK665 sensitive and resistant samples. The drug response groups were clearly distinguished by disease maturation phenotype and specific gene expression patterns. Additionally, we found that high *ABCB1* expression is a predictive indicator of MIK665 resistance in AML samples and that its co‐inhibition can induce MIK665 sensitivity in this subgroup of patients. We also identified the combination of MIK665 and venetoclax as an effective strategy to overcome resistance to either agent in AML cell lines and patient samples. This study uncovers novel response patterns to MIK665 in AML that could aid the identification of patients that would benefit from MCL‐1 inhibitor‐based therapy and improve outcomes.

## 2 Materials and methods

### 2.1 Patient samples

Bone marrow (BM) aspirates and matched skin biopsies from patients with AML (*n* = 42), as well as healthy BM control samples (*n* = 2), were collected between April 2014 and December 2017 following written informed consent, according to protocols approved by the local ethics committee (permit numbers 239/13/03/00/2010 and 303/13/03/01/2011), and in compliance with the Declaration of Helsinki. The AML patient samples were either requested from the Finnish Hematology Registry and Clinical Biobank or collected from patients cared for at the Helsinki University Hospital. BM mononuclear cells (MNCs) were isolated by Ficoll density gradient centrifugation (GE Healthcare, Buckinghamshire, UK) and suspended in conditioned medium (CM: RPMI 1640, 12.5% HS‐5 conditioned medium, 10% fetal bovine serum (FBS), 2 mm l‐glutamine, 100 units·mL^−1^ penicillin and 100 μg·mL^−1^ streptomycin) [21]. All AML samples contained at least 50% blasts. DNA and RNA from the BM MNCs were prepared using the AllPrep DNA/RNA/miRNA Universal kit or miRNeasy mini kit (Qiagen, Hilden, Germany) and then subjected to exome and RNA sequencing as previously described [22, 23]. Remaining MNCs from the samples were viably cryopreserved for further experiments. The AML patient samples' main clinical features, cytogenetics, and mutation data are detailed in Table S1 and summarized in Table 1.

**Table 1:** Clinical characteristics of the AML patient cohort. AML, acute myeloid leukemia; FAB, French‐American‐British classification; *n*, number of samples; NA, data not available.

	Total (*n* = 42)
Median age (range), years	63 (22–79)
Disease stage	
Diagnosis, *n* (%)	36 (85.7%)
Relapse, *n* (%)	4 (9.5%)
Refractory, *n* (%)	2 (4.8%)
Median blast percentage (range)	70.5 (50–95)
FAB	
AML M0/1, *n* (%)	16 (38.1%)
AML M2, *n* (%)	11 (26.2%)
AML M4, *n* (%)	3 (7.1%)
AML M5, *n* (%)	6 (14.3%)
NA, *n* (%)	6 (14.3%)

### 2.2 Drug screening setup in primary patient samples

Viably cryopreserved AML and healthy MNCs were thawed, treated with DNase I, and suspended in CM for *ex vivo* drug testing. All samples (*n* = 42) were tested with MCL‐1 inhibitors MIK665 and S63845, and BCL‐2 inhibitor venetoclax. The drugs were dissolved in DMSO and pre‐plated on 96‐well V‐bottom plates (Thermo Fisher Scientific, Waltham, MA, USA) in 7 increasing concentrations from 0.1 to 1000 nm using an Echo 550 acoustic liquid handler (Labcyte, San Jose, CA, USA). Cells were incubated on the drug plates for 48 h at 37 °C in 5% CO_2_. To filter out poor‐quality samples, each sample's baseline viability was calculated as the fraction of its live cells out of total singlets in DMSO on day 2, and only samples with a viability ≥ 20% were included in the study cohort.

Following initial profiling, selected AML samples (*n* = 15) were used for further drug combination testing of MIK665 combined with ABCB1 inhibitors elacridar or tariquidar, BCL‐2 inhibitor venetoclax, or BCL‐XL inhibitor A1331852. Out of the 15 samples, four were additional to the original cohort. For the MIK665 and ABCB1 inhibitor combination screens, each drug was tested in five increasing concentrations (MIK665: 1 to 1000 nm, elacridar/tariquidar: 10 to 5000 nm) alone or in combination with the other drug at a fixed concentration (100 nm for MIK665 and 500 nm for elacridar/tariquidar). For the MIK665 and venetoclax or A1331852 combination screens, each drug was tested in six increasing concentrations (1–1000 nm), alone or in combination with the other drug fixed at 30 nm (Fig. S1A,B). The drugs were delivered simultaneously, and the samples were incubated with the combination for 48 h at 37 °C in 5% CO_2_. All drugs used in the single agent and combination screens are summarized in Table S2.

### 2.3 Multiparametric flow cytometry‐based drug screening readout

Following drug treatments, plates were centrifuged for 6 min at 500 ***g***, and the supernatant was removed by flipping the plate. Cells were stained with CD45 and CD14 (BD Biosciences, San Diego, CA, USA) antibodies in 5% FBS in PBS (25 μL total antibody mix per well) and incubated for 30 min at room temperature in the dark (Table S1). After staining, the cells were centrifuged at 500 ***g***. This was followed by a viability‐staining step with Annexin V and 7‐AAD (BD Biosciences) to identify apoptotic and dead cells, respectively, for 10 min at room temperature in the dark. Each well was stained with 25 μL of the viability mix diluted in 1× Annexin V Binding Buffer (in ultrapure water) (Table S2). After the viability‐staining step, the plates were analyzed on the iQue Screener PLUS (Sartorius, Göttingen, Germany). The sipping parameters were 17 s per well with a pump speed of 29 r.p.m. Cell gating was performed using the forecyt software (Sartorius). An illustration of the gating strategy is available in Fig. S2.

### 2.4 Cell line expression and dependency analysis

Publicly available data were downloaded from the DepMap portal and analyzed in graphpad prism (Boston, MA, USA). Gene expression and dependency data was available for 45 and 25 myeloid leukemia cell lines, respectively. A low Chronos score corresponds to a high gene dependency.

### 2.5 Cell line culture

HEL (RRID: CVCL_0001), HL‐60 (RRID: CVCL_0002), MOLM‐13 (RRID: CVCL_2119), and Kasumi‐1 (RRID: CVCL_0589) cell lines were purchased from DSMZ (Braunschweig, Germany). MV4‐11 (RRID: CVCL_0064) cell line was purchased from ATCC (Manassas, VA, USA). All cells were maintained in RPMI 1640 medium supplemented with 10–20% heat‐inactivated FBS, l‐glutamine (2 mm), penicillin (100 U·mL^−1^), and streptomycin (100 mg·mL^−1^) at 37 °C in 5% CO_2_. All cell lines used in this study were tested to confirm that they were mycoplasma‐free and were all authenticated in the past 3 years, establishing their purity and correct identity.

Cell line authentication was performed on DNA extracted from the cells using the Promega GenePrint24 System (Promega, Madison, WI, USA). The Promega GenePrint24 system allows co‐amplification and detection of 24 human loci (22 autosomal STR loci and Amelogenin and DYS391 for gender identification). These loci collectively provide a genetic profile with a random match probability lower than 1 in 2.92 × 10^9^. The genetic profile was calculated according to the allele information found from the following sources: ATCC STR database of Human Cell Lines, JCRB STR database of Human Cell Lines, ICLC STR database of Human Cell Lines (CLIMA2.1), the DSMZ Online STR database, and the Cellosaurus resource.

### 2.6 Knockout of ABCB1 in HEL cells using CRISPR/Cas9

The gRNA targeting exon ENSE00003398270 of ABCB1 (5′ – AAGTCCAGCCCCATGGATGA – 3′) was inserted into the lentiCRISPRv2GFP vector following the protocol as previously described by Ran et al. [24]. Plasmids were purified using NucleoSpin Plasmid mini kit (Macherey‐Nagel, Düren, Germany) and the yield of each plasmid was determined using Qubit fluorometer (Thermo Fisher Scientific). HEK293‐FT cells (RRID: CVCL_6911) were seeded at a concentration of 0.5 m·mL^−1^ in a total of 10 mL of DMEM. After 24 h, cells were transfected with a second‐generation lentiviral system composed of 10 μg lentiCRISPRv2GFP‐ABCB1, 6 μg psPAX2, and 4 μg pCMV‐VSV‐G (RRIDs: Addgene_82416, Addgene_12260, and Addgene_8454, respectively) using a calcium phosphate transfection kit (Promega) with the addition of 600 μm of chloroquine. The mixture was added dropwise while swirling to homogenize. Transfections with lentiCRISPRv2GFP‐NTC or LEGO‐v2 plasmids were used as controls. 6 h after transfection, media were replaced. Lentiviral particles were harvested after 48 h incubation at 37 °C and filtered using a 0.45 μm filters.

HEL cells were seeded in a 24‐well plate at 0.2 m·mL^−1^ in 100 μL of complete RPMI. The cells were then transduced using the viral suspension added at a ratio of 4 : 1. Cells were kept in culture for 5 days at 37 °C and 5% CO_2_, and when necessary, were split 1 : 3 with a PBS wash. 1 m HEL cells were taken for fluorescence‐activated cell sorting using a BD Influx cell sorter (BD Biosciences), and GFP‐positive cells were seeded in 96‐well V‐bottom plates (Thermo Fisher Scientific) at a density of one cell per well. Cells were maintained in 100 μL of complete RPMI media per well (50 μL of fresh media and 50 μL of conditioned media from the parental cells) and expanded when needed. PCR was used to identify ABCB1 knockout, which was confirmed using Sanger sequencing and western blotting.

### 2.7 Flow cytometry‐based drug efflux testing

HEL, HEL NTC, and HEL ABCB1 KO cells were incubated with Incucyte Nuclight Rapid Red dye (Sartorius), an efflux pump substrate, at a final dilution of 1 : 1000 on a 96‐well plate [25]. 30 000 cells were added per well in a total volume of 100 μL. When assessing the effect of ABCB1 inhibition on efflux activity, elacridar was added in increasing concentrations (from 30 to 10 000 nm) alongside the Rapid Red reagent. After 24 h incubation, the plate was spun down at 500 ***g*** for 5 min and the supernatant discarded. SYTOX Blue Dead Cell Stain (Thermo Fisher Scientific) was added to the cells at a final concentration of 1 : 4000 for 10 min to assess cell viability. Plates were then analyzed by flow cytometry using the NovoCyte Quanteon (Agilent, Santa Clara, CA, USA) and gating was performed using the novoexpress software.

### 2.8 Generation of venetoclax‐resistant (VenR) cell lines

Kasumi‐1, MV4‐11, MOLM‐13, and HL‐60 cells were exposed to increasing concentrations of venetoclax (from 12.5 to 1000 nm) where the drug concentration was doubled every 2 days, as previously described [26]. VenR cell lines were derived from parental cells that continued to proliferate in the presence of 1000 nm of venetoclax.

### 2.9 CellTiter‐Glo‐based drug testing cell lines

Cell lines were tested with MIK665 and venetoclax as single agents or in combination in a range of 5 increasing concentrations from 6.25 to 100 nm using 384‐well plates (Fig. S1C). All cell lines were incubated on the drug plates at 37 °C and 5% CO_2_ for 48 h. Cell viability was measured by adding 25 μL of CellTiter‐Glo reagent (Promega) to each well, and the luminescence signal was read using a PHERAstar plate reader (BMG Labtech, Ortenberg, Germany).

### 2.10 Western blotting

Cells were lysed using RIPA buffer supplemented with phosphatase inhibitor and phenylmethylsulphonyl fluoride (Cell Signaling Technology, Danvers, MA, USA). Proteins were quantified using the Pierce BCA protein assay kit (Thermo Fisher Scientific) and subsequently separated by SDS/PAGE, then transferred to nitrocellulose membranes. Signals were acquired on an Odyssey scanner, and blots were quantified using the image studio lite software (LI‐COR Biosciences, Lincoln, NE, USA). The antibodies used are reported in Table S4.

### 2.11 Quantitative reverse transcription polymerase chain reaction (RT‐qPCR)

RT‐qPCR was performed with reference to MIQE guidelines [27]. RNA was extracted from MNCs using the NucleoSpin RNA kit (Macherey‐Nagel), quantified on a Qubit fluorometer (Thermo Fisher Scientific), and the quality assessed using an Agilent Bioanalyzer (Agilent, Santa Clara, CA, USA). cDNA was prepared using gene‐specific primers (Table S5) and SOLIScript reverse transcriptase (Solis BioDyne, Tartu, Estonia). RT‐qPCR was performed with HOT FIREPol EvaGreen qPCR Mix Plus (Solis BioDyne), using four reference genes: *SH3D19*, *HNRNPC*, *EIF4B*, and *NONO*. Reference genes were found to have low *M* values in cfx maestro software (Bio Rad, Hercules, CA, USA). Expression values per sample were normalized to the sample with the lowest expression score, and the resulting fold change values were log transformed.

### 2.12 Data analysis

For drug response assessment, viability readouts from drug‐treated wells were normalized to negative (DMSO) and positive (benzethonium chloride) controls, and inhibition dose–response curves were generated for each sample and treatment. The modified integration of these curves yielded a drug sensitivity score (DSS) [28]. The DSS is directly proportional to the response of a sample to a drug and has a range of 0 to 50. Missing values in combination matrices were imputed using Decrease, and ZIP synergy scores were calculated using synergyfinder 2.0 [29, 30].

Bulk RNA sequencing for AML patient samples was analyzed as previously described, yielding a gene‐sample raw count matrix [22, 31]. Non‐protein‐coding genes were filtered out, and Trimmed Mean of *M* values (TMM) normalization was performed using the edger package (RRID:c) and counts per million (CPM) values were computed [32]. To filter out genes with low expression across the samples, only those with a CPM value larger than 1 in at least 50% of the samples in the analysis were retained. A limma‐voom pipeline was used for log2CPM calculation and regression modeling, and differentially expressed genes were defined as those having an FDR < 0.1 [33]. Gene set enrichment analysis (GSEA) was performed using enrichr and the preranked module from genepattern [34, 35, 36, 37, 38].

Validation of results was performed using the publicly available AML patient sample datasets TCGA and BEAT AML [39, 40].

All statistical tests were performed using r software (R version 4.0.0), and nonparametric tests were used when normality of distribution by the Shapiro–Wilk test was not verified. Two group comparisons were done by the two‐sample *t*‐test or by the Mann–Whitney *U*‐test. Correlations between two continuous sets of values were performed using the Pearson or the Spearman method. Multiple testing correction by the Benjamini–Hochberg method was performed when applicable.

## 3 Results

### 3.1 Primary AML cells display variable response to MIK665

**Fig. 1:**
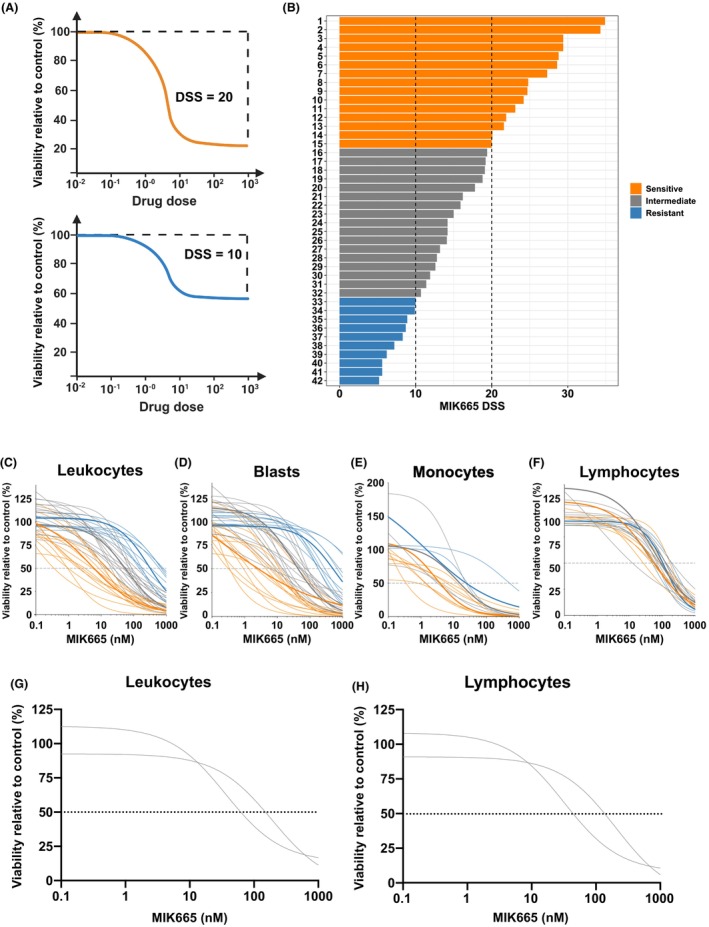
Response of AML bone marrow cell populations to MCL‐1 inhibitor MIK665. (A) An illustration showing the relationship between leukocyte cell viability dose response curves and the DSS. The DSS is a modified area over the cell viability curve, which is directly proportional to the drug sensitivity of a sample. Created with BioRender.com. (B) Waterfall plot showing the MIK665 DSS values in leukocytes of AML samples (*n* = 42) in decreasing order. DSS cutoffs were used to create sample subgroups based on response to MIK665, such that DSS ≤ 10 was considered resistant (blue), DSS ≥ 20 was considered sensitive (orange), and 10 < DSS < 20 was considered intermediate (gray). MIK665 dose–response curves for (C) leukocytes (relative IC50 values: 5.4, 45, and 134.8 nm for sensitive, intermediate, and resistant samples, respectively), (D) blast cells (relative IC50 values: 2, 44.1 and 158.4 nm respectively), (E) monocytes (IC50 values: 3.8, 14.5, and 22.9 nm respectively), and (F) lymphocytes (IC50 values: 52.6, 97.3, and 84.5 nm respectively). Representative samples with the median DSS from each group are highlighted by thicker lines on the dose–response curves. All cell populations were identified using flow cytometry. MIK665 activity was evaluated in two healthy BM samples, resulting in dose–response curves for (G) leukocytes (DSS: 10 and 12, IC50: 192 and 36.4 nm) and (H) lymphocytes (DSS: 10.7 and 14.2, IC50: 204.5 and 32.1 nm). AML, acute myeloid leukemia; BM, bone marrow; DSS, drug sensitivity score; IC50, half maximal inhibitory concentration.

Dose response of AML samples (*n* = 42) to MCL‐1 inhibitor MIK665 was assessed using a multiparametric flow cytometry‐based assay. The leukocyte cell viability dose–response curve of each sample was converted into a drug sensitivity score (DSS) directly proportional to the sample's response to MIK665 (Fig. 1A). AML leukocyte populations demonstrated a DSS continuum across the samples, ranging from 34.9 to 5.2. Based on the DSS distribution, we selected DSS cutoffs of 10 and 20 to represent resistant (*n* = 10) and sensitive (*n* = 15) samples, respectively. The remaining 17 samples were defined as intermediate responders (Fig. 1B). In parallel, S63845, an MCL‐1 inhibitor structurally related to MIK665, was also tested, resulting in a similar response pattern to MIK665 (Fig. S3) [41]. The full list of results obtained from the single‐agent screening experiments is available in Table S6, and the effect of MIK665 on the leukocytes of a sensitive and a resistant AML sample is illustrated in Fig. S4A,B. Although only a small portion of the samples were from patients with relapsed or refractory disease (*n* = 6), these had significantly lower DSS values with MIK665 compared to samples taken at diagnosis (*n* = 36) (Fig. S5A).

Viability dose–response curves for leukocyte (CD45^+^), blast (CD45dim‐sidescatter low), monocyte (CD14^+^), and lymphocyte (CD45high‐sidescatter low) subpopulations are illustrated in Fig. 1C–F. Of the 42 AML samples, 16 contained a quantifiable monocytic population (representing ≥ 5% of the total leukocytes), 75% (*n* = 12) of which were sensitive to MIK665 (Fig. 1E). Twenty‐three out of the 42 samples contained a quantifiable lymphocyte population, 96% (*n* = 22) of which were not sensitive to the treatment (Fig. 1F). Additionally, MIK665 was tested on BM MNCs from two healthy donors. Intermediate DSS values (10 and 12) were observed for the leukocyte populations, with the lymphocyte populations showing similar responses, further indicating that MIK665 has a limited effect on healthy hematopoietic cells (Fig. 1G,H). Together, these data indicated that MIK665 was particularly effective in AML cells differentiated towards the monocytic lineage, while its effect on lymphocytes was limited.

### 3.2 High expression of ABCB1 or BCL‐XL is associated with resistance to MIK665

**Fig. 2:**
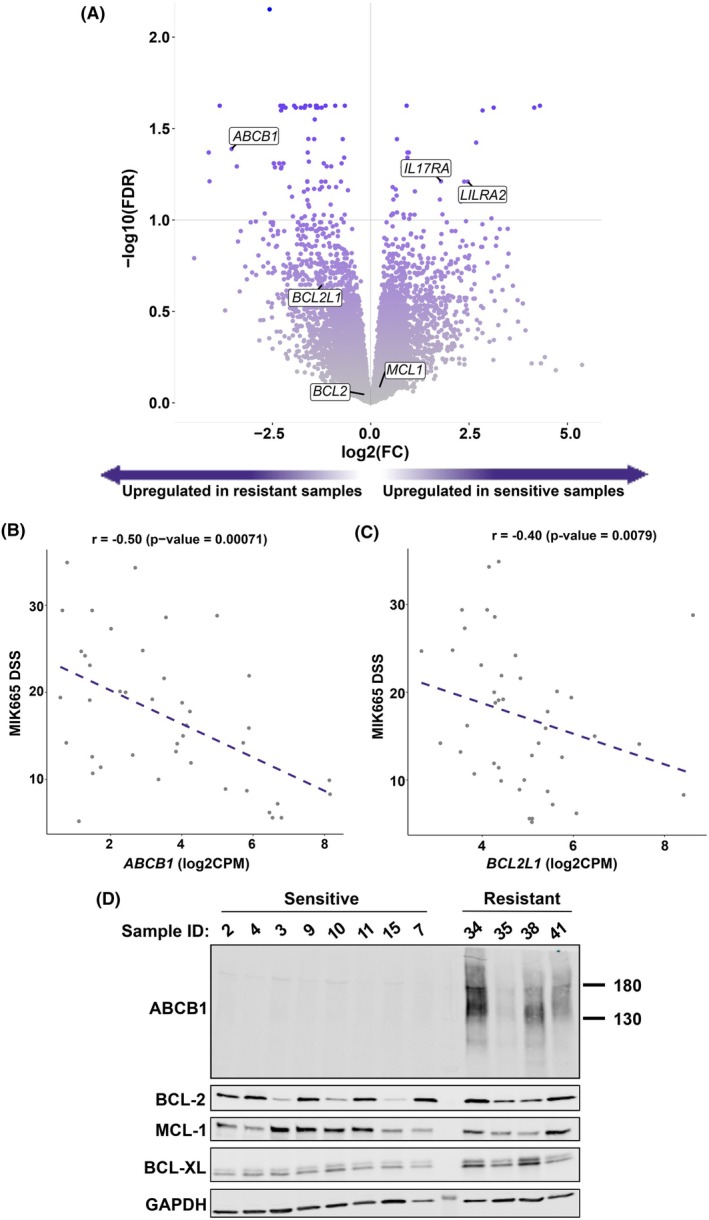
Differential gene expression analysis reveals markers of sensitivity and resistance to MIK665 in AML. (A) Volcano plot showing genes analyzed in the differential gene expression analysis. Genes to the left of the vertical line represent those upregulated in the resistant group when compared to the sensitive group, whereas those to the right of the line are downregulated genes. Genes above the horizontal line (FDR cutoff at 0.1) are significantly differentially expressed (*n* = 112). The labeled genes are *ABCB1* (log2FC = −3.54; FDR = 0.041) (enriched in the resistant samples), *LILRA2* (log2FC = 2.47; FDR = 0.062) and *IL17RA* (log2FC = 1.79; FDR = 0.061) (enriched in the sensitive samples), as well as *BCL2*, *MCL1*, and *BCL2L1*. The full list and statistics of the differentially expressed genes are available in Table S7 . (B) Scatterplot showing an inverse correlation between *ABCB1* expression (log2CPM) and MIK665 DSS in AML samples (*n* = 42; *r* = −0.5; *P* = 0.00071). (C) Scatterplot showing an inverse correlation between *BCL2L1* (log2CPM) and MIK665 DSS in AML samples (*n* = 42; *r* = −0.4; *P* = 0.0079). Correlations were performed using the Spearman method. (D) Western blot showing the protein expression levels of ABCB1, BCL‐2, MCL‐1, and BCL‐XL in a selection of MIK665‐sensitive (*n* = 8) or resistant (*n* = 4) AML samples. AML, acute myeloid leukemia; DSS, drug sensitivity score; FDR, false discovery rate; log2CPM, log2 counts per million; log2FC, log2 fold change.

To identify molecular indicators distinguishing MIK665‐sensitive (*n* = 15) and resistant (*n* = 10) samples, we performed differential gene expression analysis. The genes analyzed were restricted to protein‐coding genes having a sufficiently high expression level. As a result, 112 genes were differentially expressed (FDR < 0.1), 74 of which were upregulated and 38 were downregulated in MIK665‐resistant compared to sensitive samples (Fig. 2A, Table S7).

*ABCB1* was among the most enriched genes in the resistant samples, with a log2 fold change of −3.54 (Fig. 2A). Encoding the multidrug resistance protein MDR1, also known as P‐gp, *ABCB1* is associated with multi‐class drug resistance in a range of cancers [42]. Concordantly, results from the GSEA analysis showed an enrichment of ABC‐family‐related transport pathways, including *ABCB1*, in the resistant samples (Fig. S6A,B). *ABCB1* expression level was significantly upregulated in MIK665‐resistant samples compared to both intermediate and sensitive samples (Fig. S7A). Across the samples, *ABCB1* expression was significantly inversely correlated to MIK665 DSS, indicating that *ABCB1* expression can be used as a marker of MIK665 resistance (Fig. 2B). The difference in expression of *ABCB1* between the highest and lowest expressing samples was validated by RT‐qPCR (Fig. S8).

Of the BCL‐2 family members, *BCL2L1*, encoding the anti‐apoptotic protein BCL‐XL, had the highest expression in the resistant samples (Fig. 2A, Fig. S7B). As with *ABCB1*, we detected a significant inverse correlation between *BCL2L1* expression and MIK665 DSS (Fig. 2C). Interestingly, *BCL2L1* levels positively correlated with *ABCB1* levels in our sample cohort, a finding we confirmed in two publicly available datasets: BEAT and TCGA (Fig. S7C–E).

To confirm expression at the protein level, we performed western blotting on a set of 12 samples (8 with low *ABCB1* and 4 with high *ABCB1* RNA expression). In concordance with the transcriptomic data, elevated levels of ABCB1 and BCL‐XL were detected in MIK665‐resistant samples, but not in sensitive samples. BCL‐2 and MCL‐1 levels varied across the sensitive and resistant samples, indicating that neither was linked to primary MIK665 resistance (Fig. 2D, Fig. S9).

To determine if *ex vivo* MIK665 response was associated with any recurrently mutated genes, we analyzed somatic mutations identified by whole exome sequencing data from the tested samples. To retain commonly mutated genes in AML, only those with a mutation rate of 5% or more in the tested cohort (corresponding to ≥ 3 mutated samples out of 42) were considered for the analysis. From this analysis, we did not observe any significant associations between specific gene mutations and MIK665 response (data not shown).

Together, these results indicate that high ABCB1 and high BCL‐XL levels are indicators of MIK665 resistance and could constitute potential mechanisms of resistance to the drug in AML.

### 3.3 Differentiation markers *LILRA2* and *IL17RA* are associated with MIK665 sensitivity

Differential gene expression analysis showed *LILRA2* and *IL17RA*, encoding leukocyte immunoglobulin‐like and low affinity interleukin 17A receptors, respectively, to be among the top significantly upregulated genes in MIK665‐sensitive samples (Fig. 2A). In line with this finding, expression values of both genes were significantly correlated with MIK665 DSS values (Fig. S10A–D). Like *MCL1*, *LILRA2* and *IL17RA* are known to be associated with hematopoietic cell differentiation, notably towards monocytic and polymorphonuclear lineages (Fig. S11A–C). Since CD64 and CD14 are common monocytic markers, we also evaluated the association of these genes with MIK665 response. While *CD14* showed no association with MIK665 DSS in our cohort, *FCGR1A* (encoding CD64) expression significantly distinguished MIK665 sensitive and resistant samples and correlated with MIK665 DSS, albeit to a lower extent than *LILRA2* and *IL17RA* (Fig. S10E,F).

To study the implications of hematopoietic differentiation on MIK665 sensitivity, we compared MIK665 response levels in our cohort across differentiation subtypes based on the French‐American‐British (FAB) classification. AML samples with a more differentiated cellular phenotype (FAB M4 or M5) were more sensitive to MIK665 compared to immature samples (FAB M0/M1 or M2), even though a small subset of less differentiated samples was also responsive (Fig. S10G). These findings suggest that more differentiated AML phenotypes, characterized by high *LILRA2, IL17RA*, and *FCGR1A* expression, are more likely to be sensitive to MCL‐1 inhibition by MIK665, in line with our observation that MIK665 is more effective in monocytic populations.

### 3.4 AML cell lines with high *ABCB1* expression demonstrate distinct dependency patterns on BCL‐2 family members

**Fig. 3:**
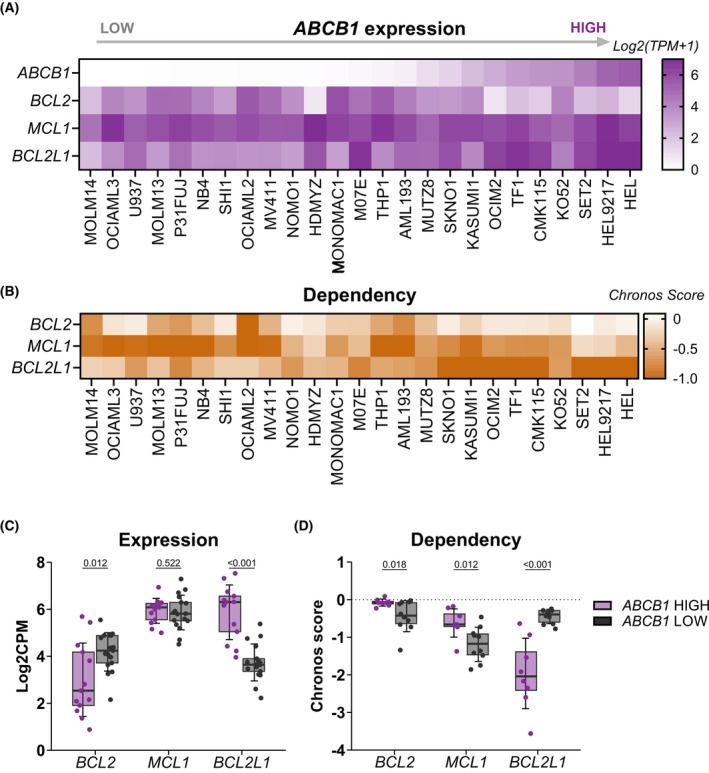
DepMap analysis of AML cell line data reveals that high *ABCB1* expression is associated with *BCL2L1* upregulation and increased dependency. (A) Heatmap showing *ABCB1*, *BCL2*, *MCL1*, and *BCL2L1* expression (log2(TPM + 1)) in AML cell lines sorted based on *ABCB1* expression. (B) Heatmap showing dependency (Chronos score) of AML cell lines on *BCL2*, *MCL1*, and *BCL2L1*. Cell line order is based on *ABCB1* expression. Boxplots representing *BCL2*, *MCL1*, and *BCL2L1* (C) expression and (D) dependency in cell lines with high (purple) and low (gray) *ABCB1* levels. log2(TPM + 1) ≥ 2 was defined as high expression of *ABCB1*, whereas log2(TPM + 1) < 2 was considered low expression. Each dot represents a single cell line. Error bars represent standard deviation. Significance was evaluated using the two‐sample *t*‐test. AML, acute myeloid leukemia; log2(TPM + 1), log2 (transcripts per million + 1).

To further investigate potential relationships between *ABCB1* and *BCL2* family anti‐apoptotic genes, we analyzed gene expression (*n* = 45) and dependency data (*n* = 25) of AML cell lines (Fig. 3A,B; Fig. S12). Cell lines were categorized as having high (log2CPM > 2) or low (log2CPM < 2) *ABCB1* expression, and the two groups were contrasted for their expression of and dependence on *BCL2*, *MCL1*, and *BCL2L1*. AML cell lines with high *ABCB1* expression had significantly reduced *BCL2* and significantly increased *BCL2L1* expression compared to the cell lines with low *ABCB1* levels, while no difference in the *MCL1* expression was detected between the two groups (Fig. 3C). Concordantly, AML cell lines with high *ABCB1* expression demonstrated significantly higher dependency on *BCL2L1*, as well as significantly reduced *BCL2* and *MCL1* dependency (Fig. 3D). Together, these findings suggest that AML cell lines exhibiting elevated *ABCB1* expression are more dependent on BCL‐XL than on MCL‐1 or BCL‐2 for survival.

### 3.5 *ABCB1* expression is a strong predictive biomarker of MIK665 resistance

**Fig. 4:**
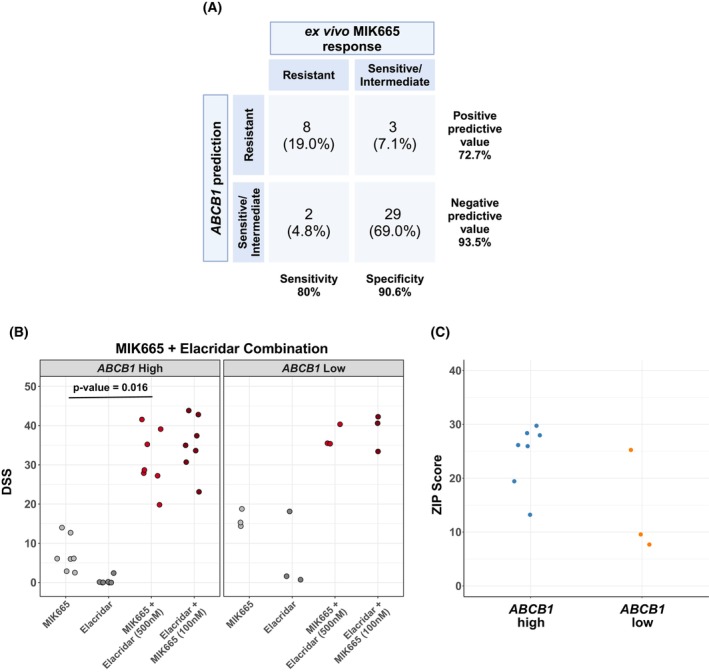
*ABCB1* expression in AML is predictive of MIK665 resistance and its co‐inhibition can induce sensitivity. (A) Contingency matrix showing performance statistics when using *ABCB1* expression at a cutoff value of log2CPM = 5.22 for MIK665 resistance prediction. Created with BioRender.com. (B) Dot plot showing the DSS of MIK665 and ABCB1 inhibitor elacridar alone or in combination in AML samples with high (log2CPM > 5.22) (*n* = 7) or low (log2CPM < 5.22) *ABCB1* expression (*n* = 3). Gray dots represent the DSS values of the single agents, whereas red dots represent the DSS values of the combination (where one drug is increased along its concentration range while the other is fixed). Significance was evaluated using the paired sample *t*‐test. (C) Synergy dot plot showing ZIP scores for the MIK665 and elacridar combination in *ABCB1* high or low samples. Responses were measured using flow cytometry following a 48 h incubation with the drugs. AML, acute myeloid leukemia; DSS, drug sensitivity score; log2CPM, log2 counts per million; ZIP, zero interaction potency.

To evaluate the predictive effect of *ABCB1* expression on MIK665 response in AML samples, we plotted the distribution of *ABCB1* expression values across the response subgroups in our cohort. The density curves of expression per subgroup showed distinct peaks, with the biggest separation difference between the resistant subgroup compared to the sensitive and intermediate subgroups (Fig. S13A). We then performed a receiver operating characteristics (ROC) curve analysis to assess the ability of *ABCB1* expression to distinguish MIK665‐resistant from sensitive and intermediate samples. The cutoff with the optimal classifying performance for *ABCB1* expression was 5.22 (log2CPM) as identified using the Youden index (Fig. S13B). This resulted in an area under the ROC curve of 0.856, with a sensitivity of 80%, a specificity of 90.6%, and a positive predictive value of 72.7% (Fig. 4A). Overall, these findings further indicate that *ABCB1* expression is a potential predictive biomarker of MIK665 resistance.

### 3.6 ABCB1 inhibition is an efficacious strategy to overcome MIK665 resistance

As ABCB1 is a known driver of multidrug resistance across several cancer types, we sought to evaluate its role in conferring resistance to MIK665 in AML. We selected seven *ABCB1* high (log2CPM > 5.22) and three *ABCB1* low samples (log2CPM < 5.22) and tested them with the ABCB1 inhibitor elacridar in combination with MIK665. It is worth noting that 500 nm was used for the fixed concentration of elacridar as this was the lowest concentration that achieved effective ABCB1 inhibition with limited effect on cell viability in the HEL cell line (Fig. S14A,B). The combination of elacridar and MIK665 resulted in significantly higher responses compared to MIK665 alone in the *ABCB1* high samples (Fig. 4B). Concordantly, synergy analysis resulted in higher ZIP synergy scores in these samples (Fig. 4C, Table S8). In parallel, we also tested a combination of MIK665 with tariquidar, another 3rd generation ABCB1 inhibitor, to rule out potential off‐target effects. Similar results were reproduced with tariquidar and MIK665, with significantly higher DSS values for the combination compared to MIK665 alone and significantly higher ZIP synergy scores in *ABCB1* high samples (Fig. S15A,B and Table S8).

To mechanistically investigate the role of ABCB1 in MIK665 resistance, we generated a HEL *ABCB1* knockout cell line using CRISPR‐Cas9 (Fig. S16A,B). The HEL cell line was selected due to its high *ABCB1* expression level and its resistance to MCL‐1 inhibition by MIK665 (Fig. 3A). However, MIK665 response in the HEL *ABCB1* knockout cells was not increased compared to HEL parental and HEL non‐target control cells (Fig. S16C). This could be due to the high dependency of HEL cells on *BCL2L1* (Chronos score = −2.6) and their low dependency on *MCL1* (−0.43), rendering MCL‐1 redundant in these cells and its inhibition ineffective (Fig. 3B). Overall, we conclude that ABCB1 expression is a resistance mechanism to MCL‐1 inhibition by MIK665, which can be overcome *ex vivo* by co‐targeting ABCB1 and MCL‐1.

### 3.7 MIK665 combined with venetoclax is an effective combination in AML samples with primary resistance to either of the single agents

**Fig. 5:**
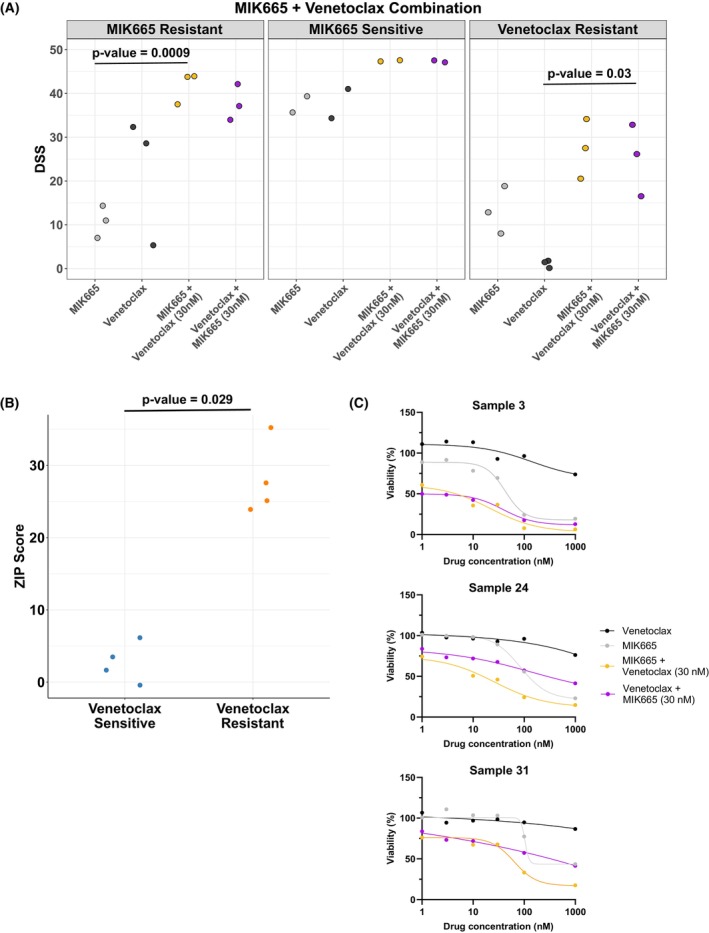
A combination of MIK665 and BCL‐2 inhibitor venetoclax is more effective compared to each agent alone and is synergistic in AML samples with primary venetoclax resistance. (A) Dot plot showing the DSS of MIK665 and venetoclax alone or in combination in MIK665‐resistant (*n* = 3), MIK665‐sensitive (*n* = 2), and venetoclax‐resistant AML samples (*n* = 3). MIK665‐resistant samples were selected as having a MIK665 DSS < 15, and MIK665‐sensitive samples were selected as having a MIK665 DSS > 30. Venetoclax‐resistant samples were selected as having a venetoclax DSS < 10. Gray dots represent the DSS values of the single agents, whereas orange or purple dots represent the DSS values of the combinations (where one drug is increased along its concentration range while the other is fixed). All responses are measured following a 48 h incubation. Significance was evaluated using the paired sample *t*‐test. (B) Synergy dot plot showing ZIP scores for the MIK665 and venetoclax combination as grouped by venetoclax response (*n* = 4). Significance was evaluated using the two sample *t*‐test. (C) Dose response curves of the venetoclax‐resistant patient samples as tested in panel A (*n* = 3). AML, acute myeloid leukemia; DSS, drug sensitivity score; ZIP, zero interaction potency.

To evaluate whether co‐targeting other anti‐apoptotic proteins is effective in MIK665‐resistant samples, we tested combinations of MIK665 with BCL‐XL inhibitor A1331852 or BCL‐2 inhibitor venetoclax in three MIK665‐resistant and three MIK665‐sensitive samples. The combination of MIK665 and A1331852 did not result in increased sensitivity of the MIK665‐resistant samples compared to treatment with MIK665 alone (Fig. S17A). However, all three MIK665‐resistant samples had significantly higher DSS values for the MIK665 and venetoclax combination compared to MIK665 alone, which suggests that targeting both MCL‐1 and BCL‐2 is an effective strategy to overcome resistance to MIK665 (Fig. 5A).

Interestingly, we noticed that one of the MIK665‐resistant samples that responded well to the combination was also *ex vivo* primary resistant to venetoclax. To similarly assess the potential of co‐targeting anti‐apoptotic proteins in the context of primary venetoclax resistance, we tested three additional samples with primary venetoclax resistance with the MIK665 and venetoclax or A1331852 combinations (Fig. S3, Table S6). The combination of MIK665 and venetoclax showed significantly higher DSS values in all three venetoclax‐resistant samples compared to venetoclax alone, as well as significantly higher ZIP synergy scores (Fig. 5B,C and Table S8). Interestingly, the combination of MIK665 and A1331852 was also significantly more synergistic in venetoclax‐resistant samples (Fig. S17B). Together, these results demonstrate that a combination of MIK665 and venetoclax is effective in overcoming primary resistance to either of the two agents, and that a combination of MIK665 and A1331852 is also effective in primary venetoclax‐resistant samples.

### 3.8 MIK665 combined with venetoclax restores sensitivity in AML cell lines with acquired venetoclax resistance

**Fig. 6:**
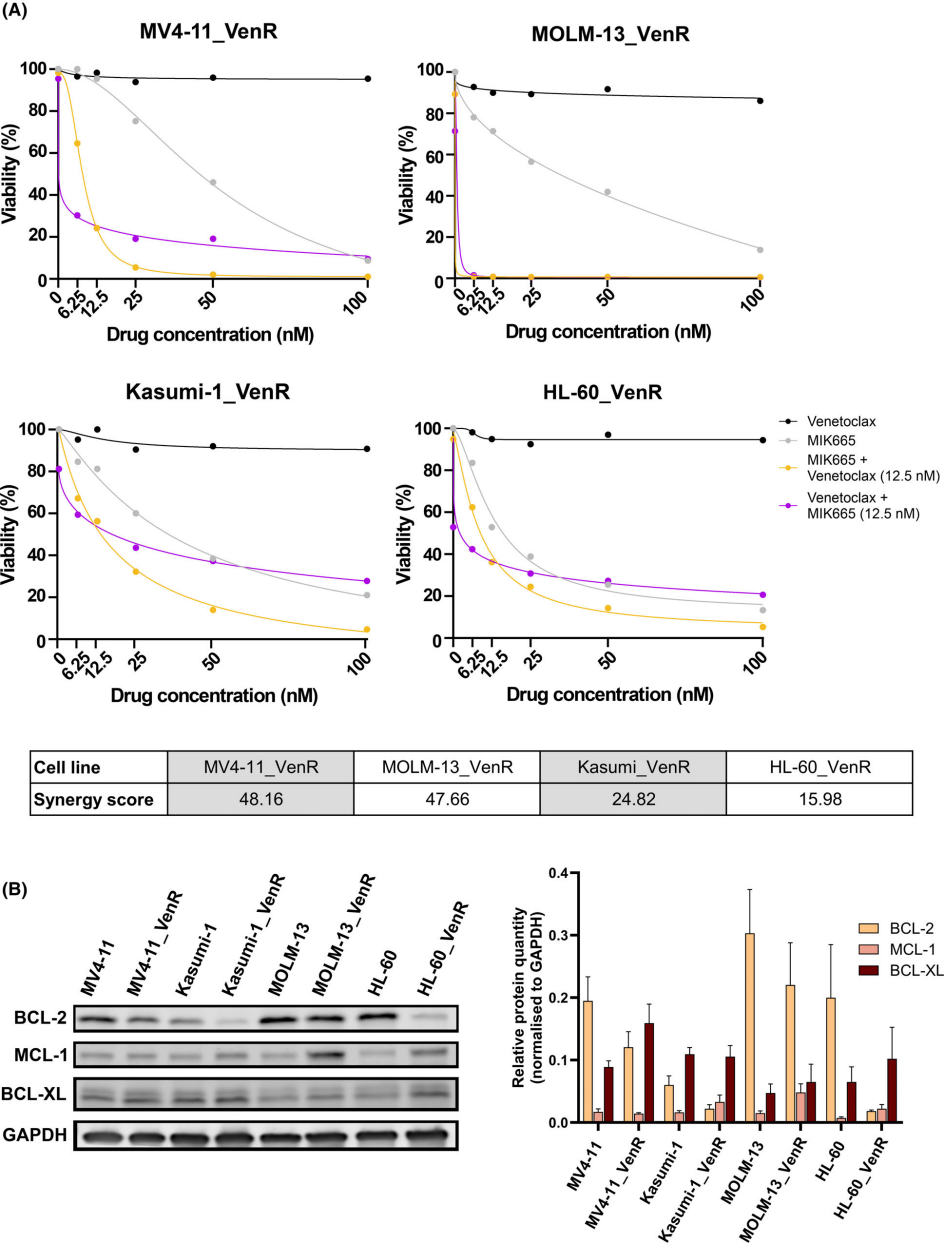
A combination of MIK665 and venetoclax is synergistic in AML cell lines with acquired resistance to venetoclax. (A) Dose–response curves of MIK665 and venetoclax alone and in combination in MV4‐11 venetoclax‐resistant (MV4‐11_VenR), MOLM‐13 venetoclax‐resistant (MOLM‐13_VenR), Kasumi‐1 venetoclax‐resistant (Kasumi‐1_VenR), and HL‐60 venetoclax‐resistant (HL‐60_VenR) cell lines, where one drug is increased along its concentration range while the other is fixed at 12.5 nm (*n* = 1). Responses are measured by CellTiter‐Glo following a 48 h incubation. ZIP synergy scores for the combination in each of the cell line are reported in the table. (B) Western blot showing the protein expression levels of BCL‐2, MCL‐1, and BCL‐XL in parental (*n* = 4) and VenR (*n* = 4) cell lines, and the protein quantification normalized to the respective GAPDH levels (*n* = 3). Error bars represent the standard error of mean. AML, acute myeloid leukemia; VenR, venetoclax‐resistant; ZIP, zero interaction potency.

As acquired resistance to venetoclax is commonly observed following venetoclax‐based treatment, we tested the MIK665 and venetoclax combination in four pairs of AML cell lines prior to and following acquired venetoclax resistance. We generated the VenR cell lines MV4‐11_VenR, Kasumi‐1_VenR, MOLM‐13_VenR, and HL‐60_VenR by exposing the parental cell lines to increasing concentrations of venetoclax. Out of the parental cell lines, MV4‐11 and MOLM‐13 had low ZIP synergy scores with the MIK665 and venetoclax combination since they were baseline sensitive to the drugs as single agents. On the other hand, HL‐60 and Kasumi‐1 were baseline resistant to either or both single agents and therefore displayed higher synergy scores (Fig. S18). In all VenR cell lines, MIK665 in combination with venetoclax showed high ZIP synergy scores, with a greater effect in MV4‐11_VenR and MOLM‐13_VenR than in Kasumi‐1_VenR and HL‐60_VenR (Fig. 6A).

Comparison of BCL‐2 family protein levels between parental and VenR cell lines showed a clear drop in BCL‐2 expression and a trend toward increasing BCL‐XL and MCL‐1 expression upon acquired venetoclax resistance (Fig. 6B). These findings show that MIK665 restores sensitivity to venetoclax in AML cell lines with acquired venetoclax resistance, suggesting that this combination can have clinical relevance upon patient relapse to venetoclax‐based treatment regimens.

## 4 Discussion

Our assessment of MCL‐1 inhibitor MIK665 activity in primary AML patient samples resulted in varied responses, reflecting the heterogeneous response observed in clinical trials [16, 17]. *ABCB1* was among the most significantly upregulated genes in the MIK665‐resistant samples, with logistic regression and ROC curve analyses revealing its strong performance in identifying MIK665‐resistant samples. Targeting ABCB1 in combination screens using third‐generation ABCB1 inhibitors elacridar and tariquidar was effective at sensitizing resistant samples to MIK665 *ex vivo*. Additionally, co‐inhibition of MCL‐1 and BCL‐2 by the addition of venetoclax proved to be another effective strategy in the MIK665‐resistant population.

ABCB1, commonly referred to as multidrug resistance protein 1 (MDR1) or P‐glycoprotein (P‐gp), can confer resistance to several cancer therapeutics [43]. In AML, the chemotherapeutic daunorubicin is a known ABCB1 substrate, and high *ABCB1* expression is more prevalent in adverse European LeukemiaNet (ELN) risk patients having poor outcomes when treated with standard chemotherapy [44, 45, 46]. Previous research in multiple myeloma has demonstrated that ABCB1‐mediated drug efflux also represents a resistance mechanism to MCL‐1 inhibitors, which can be overcome following concomitant inhibition of ABCB1 [47]. To determine whether ABCB1 inhibition can overcome MIK665 resistance in AML, we evaluated the efficacy of a combination of MIK665 and third‐generation ABCB1‐specific inhibitors elacridar and tariquidar in samples with high *ABCB1* expression and low response to MIK665. The combinations were synergistic and more effective at eliminating AML cells in patient samples compared to MIK665 alone. It is worth noting that the observed synergy for the combinations occurred at relatively high concentrations of the ABCB1 inhibitors, which may be challenging to achieve clinically. This is supported by the fact that despite efforts to investigate ABCB1 inhibitors in clinical trials, progress has been challenged notably by limited clinical efficacy and unfavorable toxicity profiles [46, 48, 49]. This highlights the need for development of novel ABCB1 inhibitors with improved pharmacokinetic and safety profiles. Together, these findings indicate that *ABCB1* expression represents a predictive biomarker and a potential resistance mechanism to MIK665.

Interestingly, relapse and refractory samples in our cohort were significantly more resistant to MIK665 than samples taken at diagnosis. However, this was not associated with elevated *ABCB1* expression, possibly due to the limited number of relapse/refractory samples in our cohort (Fig. S5B). This warrants further evaluation of the role of ABCB1 in the context of relapsed and refractory AML in larger studies.

In contrast, samples in our cohort with higher expression levels of *LILRA2, ILI7RA*, and *FCGR1A* (encoding CD64), markers of phenotypically mature monocytic‐like AML subtypes (notably M4 and M5), were more sensitive to MCL‐1 inhibition by MIK665 [50]. These results align with earlier studies demonstrating that AML FAB subtypes M4 and M5 have higher expression of and dependence on MCL‐1 and are thus sensitive to MCL‐1 inhibition [51, 52]. As expected, M4 and M5 samples in our cohort also harbored lower expression levels of *ABCB1* compared to less differentiated FAB types (M0, M1 and M2), a finding replicated in the BEAT and TCGA AML patient cohorts (Fig. S19A–F, Table S9).

Previous research has reported that high levels of the anti‐apoptotic protein BCL‐XL correlate with resistance to MCL‐1 inhibition [13, 41, 53], a finding also observed in this study's MIK665‐resistant patient samples. Interestingly, *BCL2L1*, the gene encoding BCL‐XL, strongly correlated with *ABCB1* expression in three AML patient sample cohorts and demonstrated a similar expression trend across FAB types (Figs. S7 and S19, Table S9). AML cell line analysis further demonstrated that high *ABCB1* expression had significantly higher expression of and dependence on *BCL2L1*. Kuusanmäki et al. found that high BCL‐XL expression was correlated with sensitivity to BCL‐XL inhibitors in erythroleukemia (FAB M6/M7) [34]. However, in our study, MIK665‐resistant samples did not respond to the BCL‐XL inhibitor A1331852 alone or in combination with MIK665, likely due to our study cohort lacking samples with M6/M7 phenotypes. Further studies are needed to elucidate the mechanistic link between *ABCB1* expression and *BCL2L1* dependency, notably in phenotypically undifferentiated AML subtypes.

*In vitro* studies have indicated that MCL‐1 inhibitors synergize with BCL‐2 inhibitors, namely venetoclax, leading to the evaluation of this combination for AML patients in several clinical trials [11, 13, 17]. Aligning with these observations, we found that the combination of MIK665 and venetoclax is effective in samples with MIK665 primary resistance. Interestingly, this combination was also effective and synergistic in AML samples with primary venetoclax resistance, a finding with particular clinical relevance as up to 30% of AML patients treated with venetoclax‐based regimens are found to be primary refractory to the treatment [2, 54, 55, 56, 57]. Clinical trials have reported cases of disease progression and acquired resistance to venetoclax‐based therapies following initial remissions in over 50% of patients, leaving them with limited treatment options and a dismal prognosis [56, 58]. Using AML cell lines with acquired venetoclax resistance as a model for clinical venetoclax relapse, we observed that the MIK665 and venetoclax combination was also highly effective in this context. This is in line with previous findings showing that MCL‐1 upregulation contributes to acquired venetoclax resistance, and that the addition of an MCL‐1 inhibitor has the potential to restore sensitivity [59, 60]. Overall, the combination of MIK665 and venetoclax was effective in AML patient samples with primary resistance to either of the single agents, as well as AML cell lines with acquired venetoclax resistance, a finding warranting further clinical evaluation. The elevated sensitivity of such AML patient subgroups to the combination of MIK665 and venetoclax suggests that lower treatment doses could be administered, thereby improving toxicity profiles, which have so far represented a major obstacle for MCL‐1 inhibitor development.

## 5 Conclusion

In summary, the associations between diverse molecular features of AML samples and their sensitivity to MIK665 described in this study provide valuable insights from a precision medicine and clinical trial design viewpoint. This is of particular relevance to the ongoing clinical evaluation of MCL‐1 targeting drugs, notably the MIK665‐based antibody drug conjugate S227928 [18, 19]. Our study identifies a sizable target AML patient population with differentiated disease that is susceptible to MCL‐1 inhibition by MIK665. Moreover, we show that elevated *ABCB1* expression represents a predictive biomarker and potential mechanism of resistance to MIK665, which can be overcome *ex vivo* by the addition of an ABCB1 specific inhibitor, or by co‐targeting BCL‐2, a finding with clinical relevance. Our findings support the evaluation of the combination of MIK665 with venetoclax in resistant patient populations to restore and prolong clinical responses.

## Conflict of interest

SA is currently an employee of Faron Pharmaceuticals, and ED is an employee of Owkin. During their participation in this study, ED, JW, HM, and EH were employees and shareholders of Novartis. CAH received research funding from Novartis related to this work and has received unrelated research funding from BMS/Celgene, Kronos Bio, Oncopeptides, Orion Pharma, WntResearch, and the Innovative Medicines Initiative 2 project HARMONY, plus personal fees from Amgen and Autolus. MK has received personal fees from Astellas Pharma, AbbVie, Bristol‐Myers Squibb, Faron Pharmaceuticals, Novartis, and Pfizer outside the submitted work. KP has received unrelated research funding from Incyte, Novartis, and Roche.

## Author contributions

CAH and EH conceived and supervised the study. HM and JW contributed to the conception and design of the study. JS, RN, EK, SA, MT, HK, AP, JJM, KKJ, and EJO designed and performed the experimental work. JS, RN, EK, ED, and NI carried out the data analysis and interpretation. MK and KP collected the samples and interpreted clinical data. JS and RN wrote the manuscript, and all authors critically read the manuscript, provided constructive comments, and agreed to the content. CAH provided infrastructure to carry out the work.

## Supporting information


**Fig. S1.** Schematic of the drug combination tested with primary patient samples and cell lines.
**Fig. S2.** Gating strategy.
**Fig. S3.** Heatmap summarizing the response of the AML sample cohort to MCL‐1 inhibitors MIK665 and S63845, and to BCL‐2 inhibitor venetoclax.
**Fig. S4.** Representative dot plots showing the response of a sensitive and a resistant AML sample to MIK665.
**Fig. S5.** Comparison of diagnosis and relapse or refractory (R/R) primary AML samples.
**Fig. S6.** Results from the gene set enrichment analysis performed in Enrichr and GenePattern.
**Fig. S7.** *ABCB1* and *BCL2L1* have higher gene expression levels in the MIK665‐resistant sample subgroup.
**Fig. S8.** RT‐qPCR experiments verify RNA sequencing data.
**Fig. S9.** Quantification of western blots measuring ABCB1, BCL‐2, MCL‐1, and BCL‐XL levels in MIK665‐sensitive (*n* = 8) and resistant (*n* = 4) patient samples.
**Fig. S10.** Differentiation‐associated genes correlate with MIK665 sensitivity.
**Fig. S11.** Hematopoietic trees demonstrating the increasing expression of *MCL1*, *LILRA2*, and *IL17RA*.
**Fig. S12.** Heatmap showing *ABCB1*, *BCL2*, *MCL1* and *BCL2L1* expression in 45 AML cell lines.
**Fig. S13.** *ABCB1* gene expression can distinguish MIK665‐resistant AML samples.
**Fig. S14.** Flow cytometry‐based drug efflux assay to assess the activity of increasing doses of elacridar in HEL cells.
**Fig. S15.** Results of the MIK665 and tariquidar combination testing in AML patient samples.
**Fig. S16.** ABCB1 activity and MIK665 response in HEL ABCB1 knockout cells.
**Fig. S17.** Results of the MIK665 and A1331852 combination testing in AML patient samples.
**Fig. S18.** Results of MIK665 and venetoclax combination testing in parental cell lines.
**Fig. S19.** Expression of *ABCB1* and *BCL2L1* across FAB types in primary AML samples.

**Table S1.** Sample‐level annotations including clinical characteristics and common cytogenetic and mutation data.
**Table S2.** Small molecule inhibitors tested *ex vivo* in the AML sample cohort.
**Table S3.** Flow cytometry antibody panel.
**Table S4.** Antibodies used in the western blotting assay.
**Table S5.** Primer sequences used in the RT‐qPCR assay.
**Table S6.** Flow cytometry‐based drug sensitivity testing results from the single agent screens in AML patient samples (*n* = 42).
**Table S7.** List of differentially expressed genes (*n* = 112) sorted by increasing logFC.
**Table S8.** ZIP synergy scores for the combinations of MIK665 + ABCB1 inhibitor (elacridar/tariquidar), MIK665 + BCL2 inhibitor (venetoclax), and MIK665 + BCL‐XL inhibitor (A‐1331852).
**Table S9.** List of one‐way ANOVA and Tukey tests showing statistically significant differences in *ABCB1* or *BCL2L1* expression across FAB types.

## Data Availability

The drug sensitivity testing results that support the findings of this study are available in the Supporting Information of this article (Table S6). The RNA sequencing data supporting the findings of this study is available on request from the corresponding author. This data is not publicly available due to privacy or ethical restrictions.
