# Supplementary material for: Predictors of response and rational combinations for the novel MCL‐1 inhibitor MIK665 in acute myeloid leukemia
Source: Mol Oncol. 2025 Sep 21;20(2):389–408. doi: 10.1002/1878-0261.70130 (PMC12936425; doi:10.1002/1878-0261.70130)
Supplement: Supplementary file 1 — Fig. S1. Schematic of the drug combination tested with primary patient samples and cell lines. Fig. S2. Gating strategy. Fig. S3. Heatmap summarizing the response of the AML sample cohort to MCL‐1 inhibitors MIK665 and S63845, and to BCL‐2 inhibitor venetoclax. Fig. S4. Representative dot plots showing the response of a sensitive and a resistant AML sample to MIK665. Fig. S5. Comparison of diagnosis and relapse or refractory (R/R) primary AML samples. Fig. S6. Results from the gene set enrichment analysis performed in Enrichr and GenePattern. Fig. S7. ABCB1 and BCL2L1 have higher gene expression levels in the MIK665‐resistant sample subgroup. Fig. S8. RT‐qPCR experiments verify RNA sequencing data. Fig. S9. Quantification of western blots measuring ABCB1, BCL‐2, MCL‐1, and BCL‐XL levels in MIK665‐sensitive (n = 8) and resistant (n = 4) patient samples. Fig. S10. Differentiation‐associated genes correlate with MIK665 sensitivity. Fig. S11. Hematopoietic trees demonstrating the increasing expression of MCL1, LILRA2, and IL17RA. Fig. S12. Heatmap showing ABCB1, BCL2, MCL1 and BCL2L1 expression in 45 AML cell lines. Fig. S13. ABCB1 gene expression can distinguish MIK665‐resistant AML samples. Fig. S14. Flow cytometry‐based drug efflux assay to assess the activity of increasing doses of elacridar in HEL cells. Fig. S15. Results of the MIK665 and tariquidar combination testing in AML patient samples. Fig. S16. ABCB1 activity and MIK665 response in HEL ABCB1 knockout cells. Fig. S17. Results of the MIK665 and A1331852 combination testing in AML patient samples. Fig. S18. Results of MIK665 and venetoclax combination testing in parental cell lines. Fig. S19. Expression of ABCB1 and BCL2L1 across FAB types in primary AML samples. [file MOL2-20-389-s002.docx]

**Predictors of Response and Rational Combinations for the Novel MCL-1 Inhibitor MIK665 in Acute Myeloid Leukemia**

**Supplementary Figures**

**
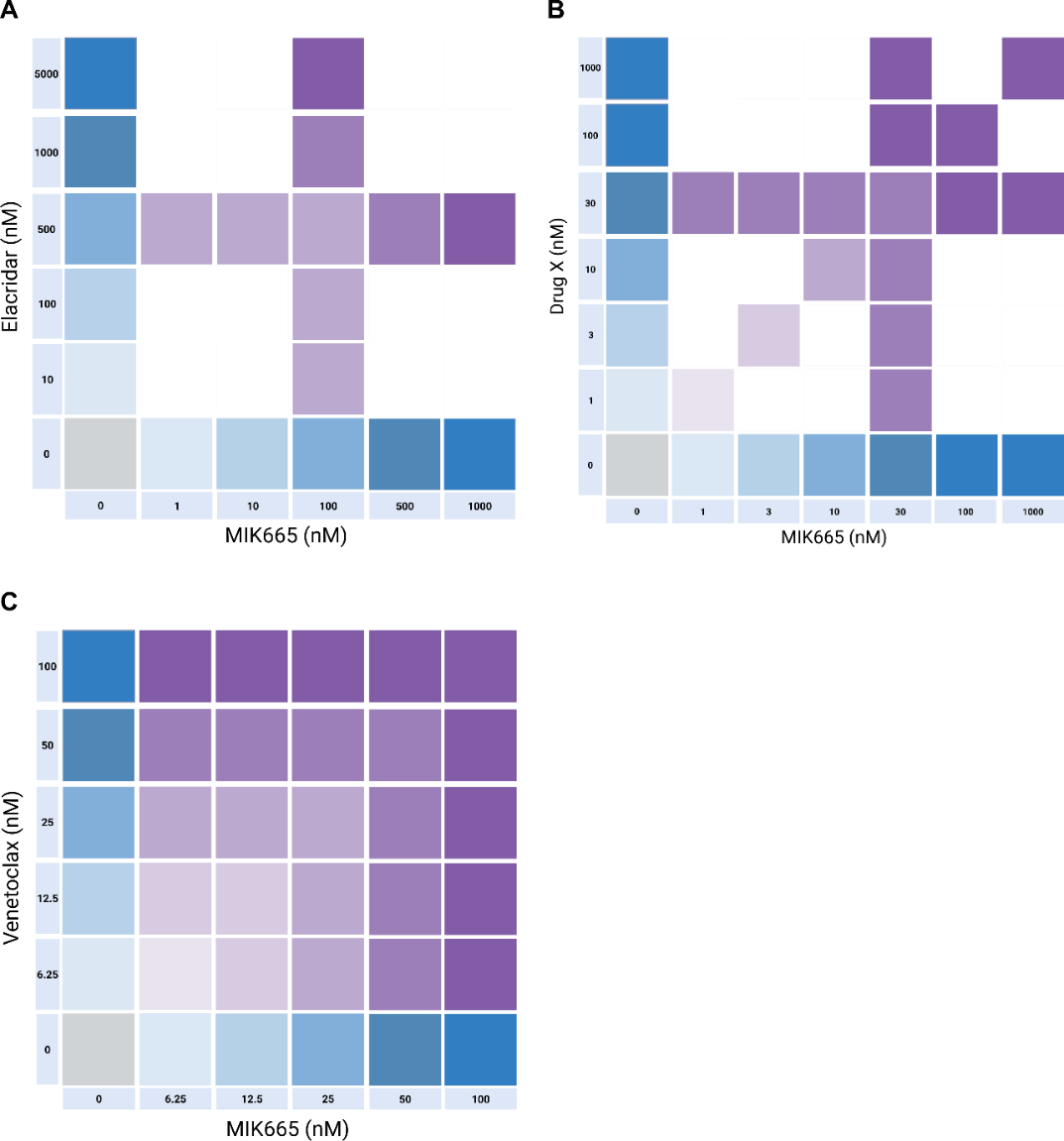
**

**Supplementary Figure 1. Schematic of the drug combination tested with primary patient samples and cell lines.** Combination of MIK665 (1 to 1000nM) and **A)** elacridar (or tariquidar) or **B)** venetoclax or A1331852 tested in patient samples. **C)** Combination of MIK665 and venetoclax tested in MV4-11, MOLM-13, Kasumi-1, and HL-60 parental and venetoclax-resistant cell lines. Colored squares in each matrix indicate the concentrations tested. Created with BioRender.com.


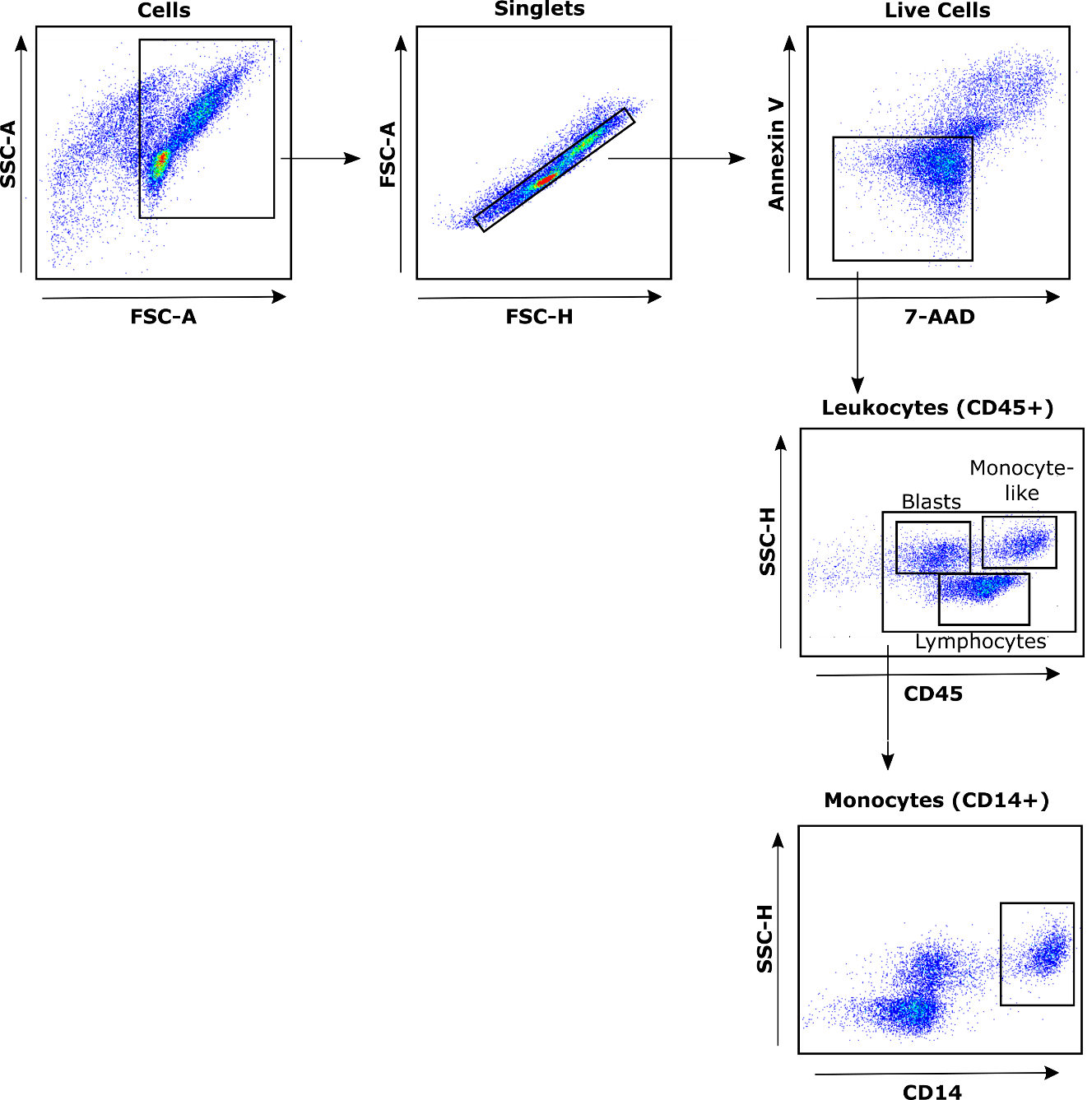


**Supplementary Figure 2. Gating strategy.** The flow cytometry gating strategy used to distinguish different constituent cell types in the AML patient sample cohort. SSC-A, side scatter-area; SSC-H, side scatter-height; FSC-A, forward scatter-area; FSC-H, forward scatter-height; AML, acute myeloid leukemia.


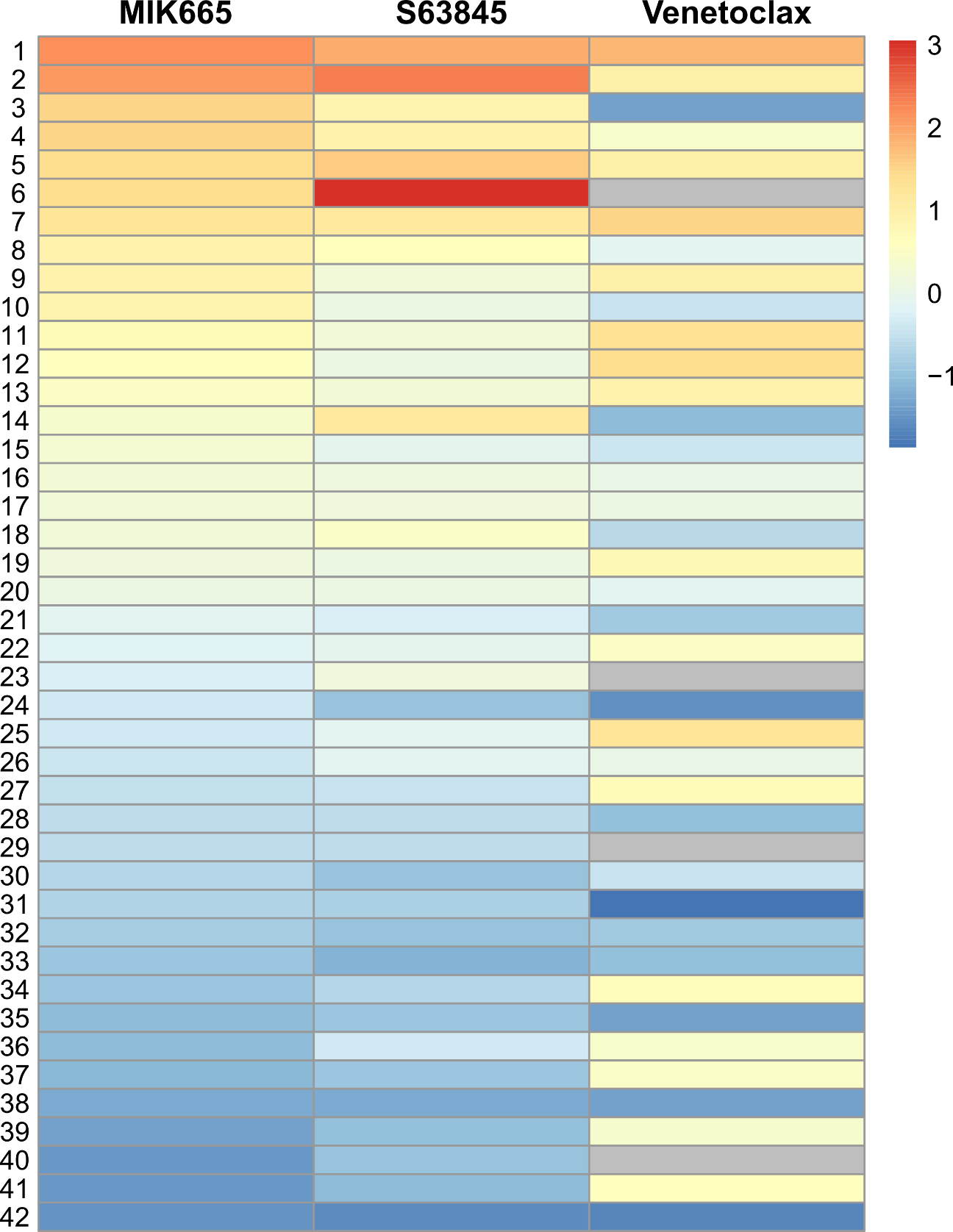


**Supplementary Figure 3. Heatmap summarizing the response of the AML sample cohort to MCL-1 inhibitors MIK665 and S63845, and to BCL-2 inhibitor venetoclax.** The DSS values obtained for the leukocyte populations were scaled and centered by drug, with low DSS values (blue) corresponding to drug resistance and high DSS values (red) corresponding to drug sensitivity (*n* = 42). Samples are ordered on the y-axis by MIK665 sensitivity. Grey boxes indicate missing or censored values. AML, acute myeloid leukemia; DSS, drug sensitivity score.


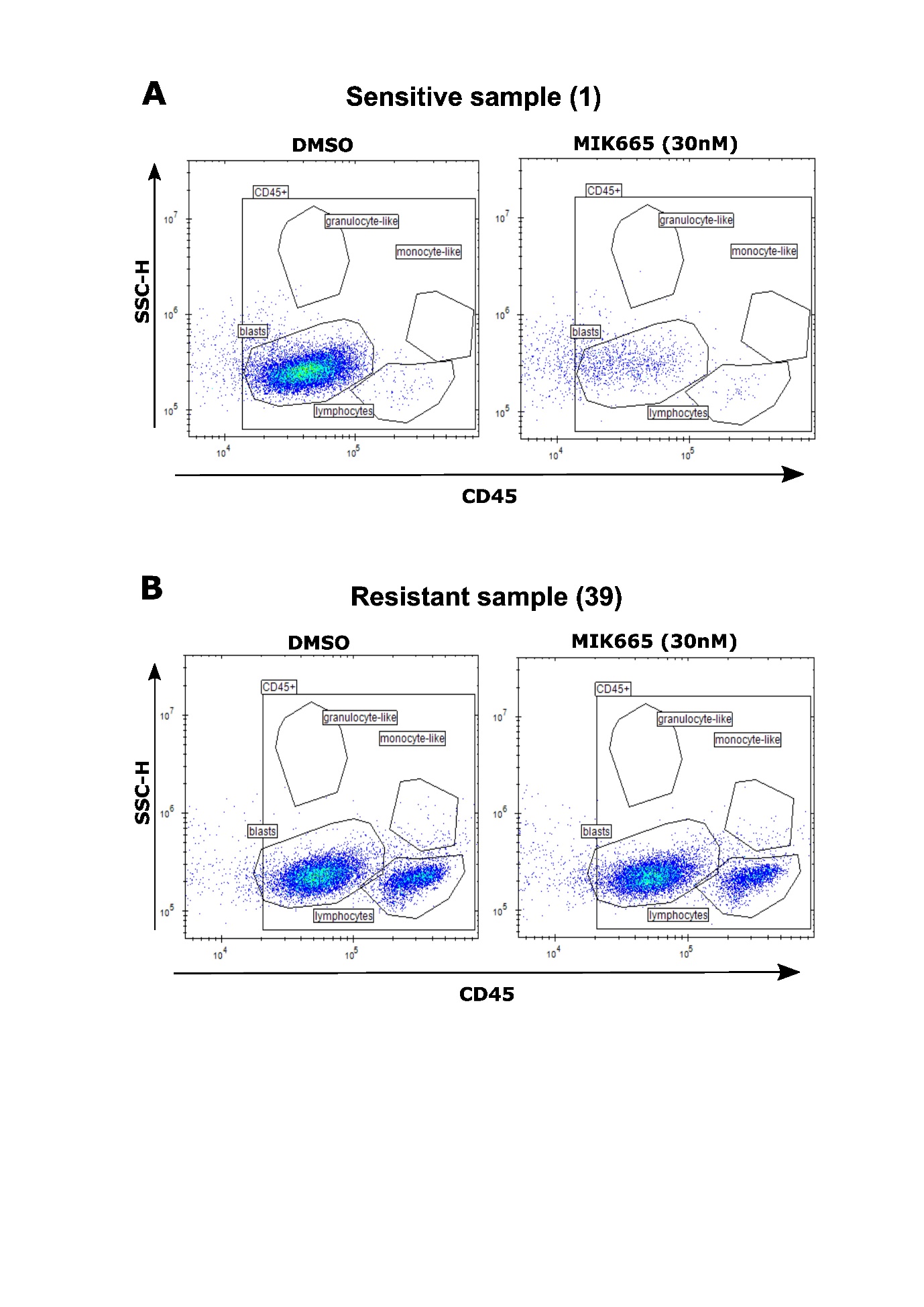
**Supplementary Figure 4. Representative dot plots showing the response of a sensitive and a resistant AML sample to MIK665. A)** A sensitive sample (sample ID:1) shows a decrease in blast cells upon MIK665 treatment when compared with the DMSO control. **B)** A resistant sample (sample ID: 39) shows no change in blast cells upon MIK665 treatment when compared to DMSO, indicating the resistance of the sample to the treatment. Following 48 h incubation with MIK665 (30 nM), samples were analyzed using multi-parametric flow cytometry. SSC-H, side scatter-height; AML, acute myeloid leukemia.


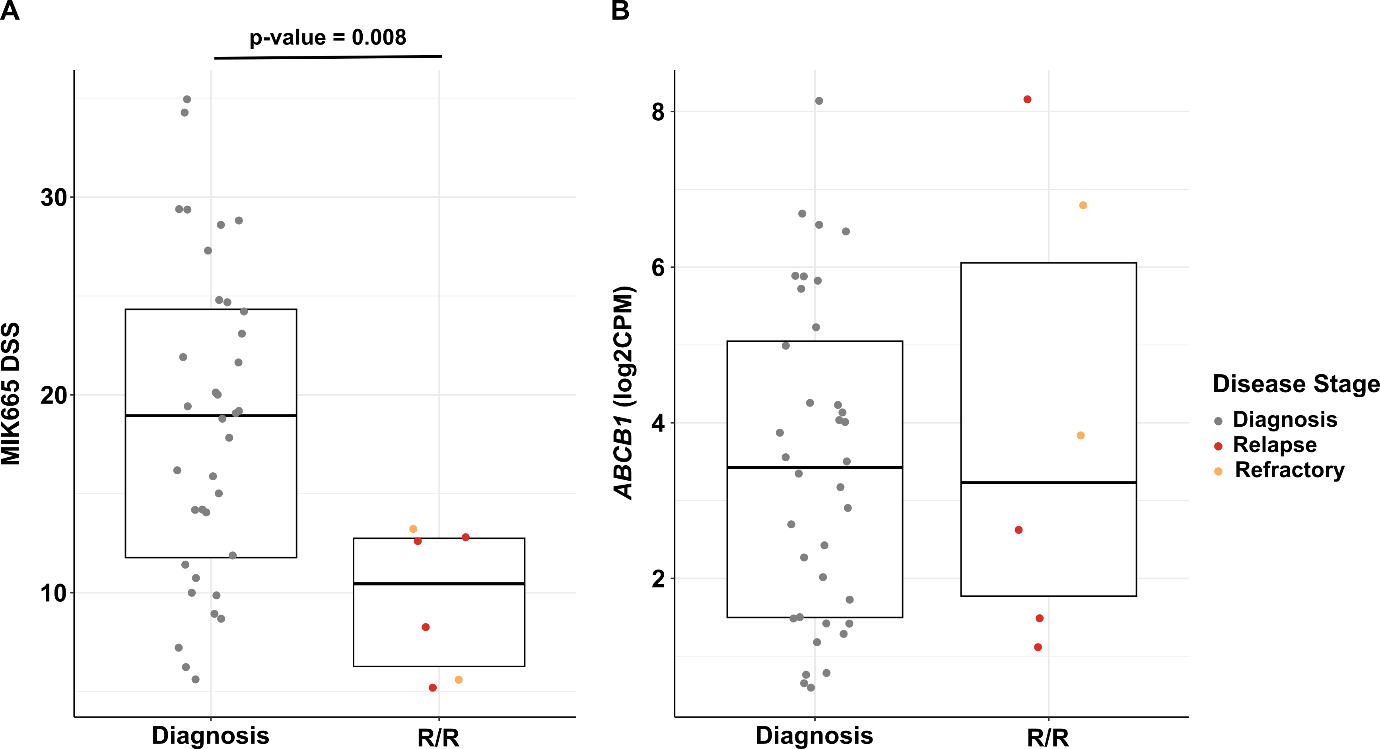


**Supplementary Figure 5. Comparison of diagnosis and relapse or refractory (R/R) primary AML samples.** Boxplots comparing the **A)** MIK665 DSS and **B)** *ABCB1* expression by the samples’ disease stage (diagnosis, *n* = 36; R/R, *n* = 6). Significance was evaluated using the Mann-Whitney U test. AML, acute myeloid leukemia; DSS, drug sensitive score; log2CPM, log2 counts per million; R/R, relapse/refractory.


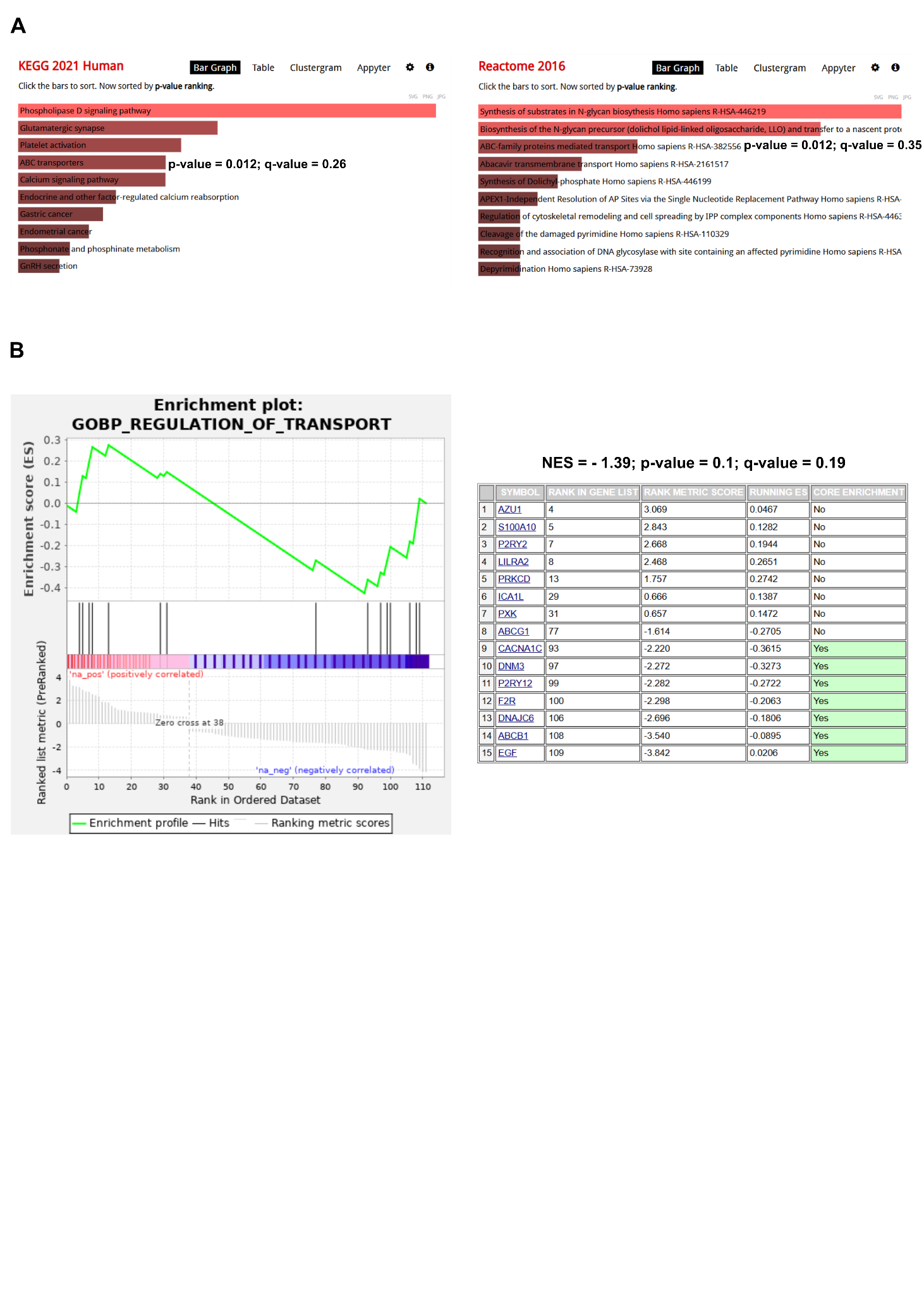


**Supplementary Figure 6. Results from the gene set enrichment analysis performed in Enrichr and GenePattern.** GSEA performed in **A)** EnrichR using genes upregulated in MIK665-resistant samples and **B)** GenePattern using the ranked list of differentially expressed genes between MIK665-sensitive and resistant samples show a significant enrichment of ABC-family mediated drug transport in the resistant samples. KEGG, Kyoto encyclopedia of genes and genomes; ES, enrichment score; NES, normalized enrichment score; GOBP, gene ontology biological process; GSEA, gene set enrichment analysis.


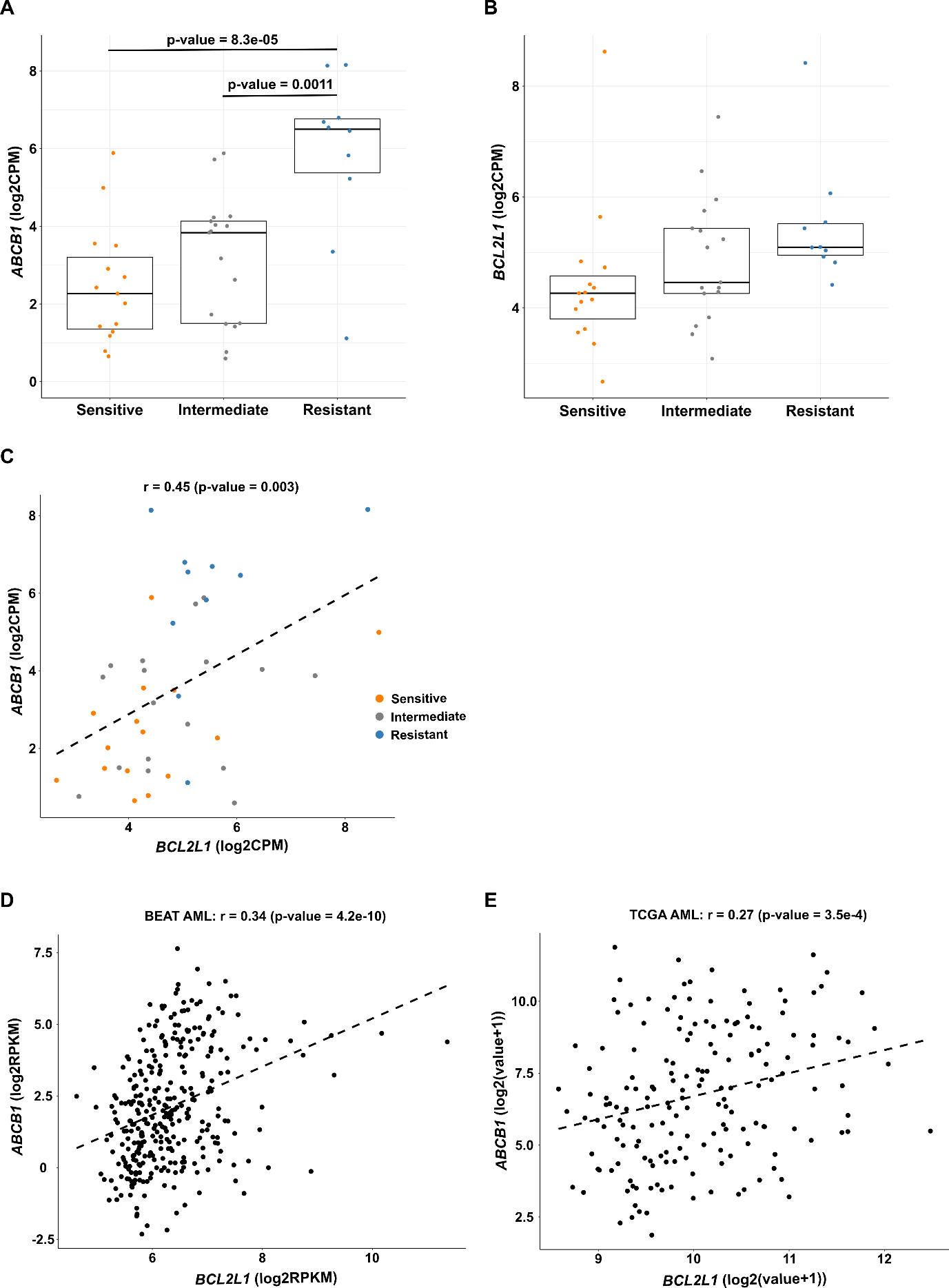


**Supplementary Figure 7. *ABCB1* and *BCL2L1* have higher gene expression levels in the MIK665-resistant sample subgroup. A)** The median expression level of *ABCB1* increases significantly with MIK665 resistance (anova p-value = 8.4E-5), and it is significantly higher in resistant samples (*n* = 10) compared to sensitive (*n* = 15) and intermediate (*n* = 17) MIK665 response groups, by the Tukey test. **B)** The median expression level of *BCL2L1* shows an increasing trend from MIK665-sensitive to resistant samples. *ABCB1* and *BCL2L1* expressions correlate positively and significantly in primary patient samples from the **C)** FIMM (*n* = 42), **D)** BEAT (*n* = 326), and **E)** TCGA (*n* = 173) AML cohorts, using the Person correlation method. Log2CPM, log2 counts per million; log2RPKM, log2 reads per kilobase per million mapped reads; FIMM, Institute for Molecular Medicine Finland; TCGA, The Cancer Genome Atlas; AML, acute myeloid leukemia.


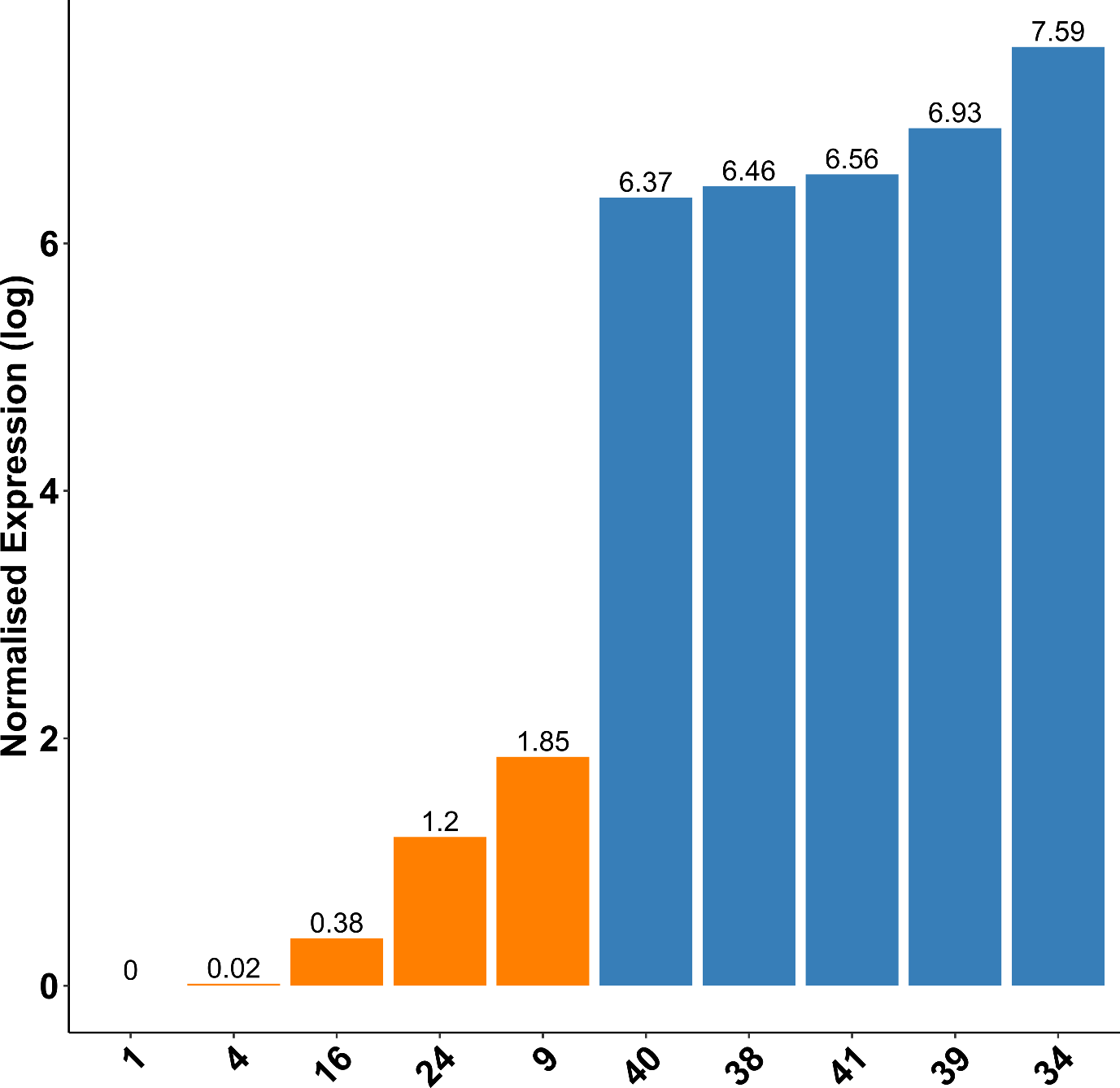


**Supplementary Figure 8. RT-qPCR experiments verify RNA sequencing data.** To validate *ABCB1* expression levels obtained from RNA sequencing data, confirmatory RT-qPCR was performed on 10 primary patient samples: 5 with low (orange) and 5 with high (blue) *ABCB1* expression. The computed normalized log fold-change ratios per sample are shown above each bar. The housekeeping genes used were *SH3D19*, *NONO*, *HNRNPC*, *EIF4B*. RT-qPCR, quantitative reverse transcription polymerase chain reaction.


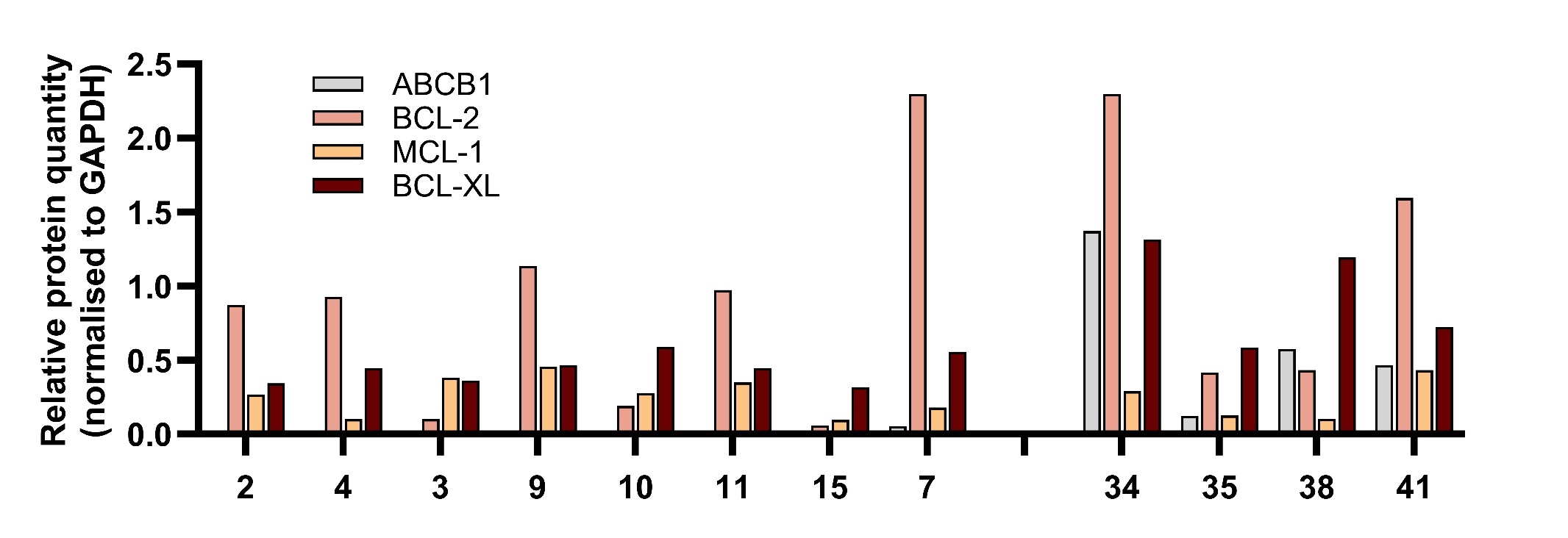


**Supplementary Figure 9. Quantification of western blots measuring ABCB1, BCL-2, MCL-1, and BCL-XL levels in MIK665-sensitive (*n* = 8) and resistant (*n* = 4) patient samples.** The relative protein quantity for each sample is normalized to GAPDH protein expression (*n* = 1).


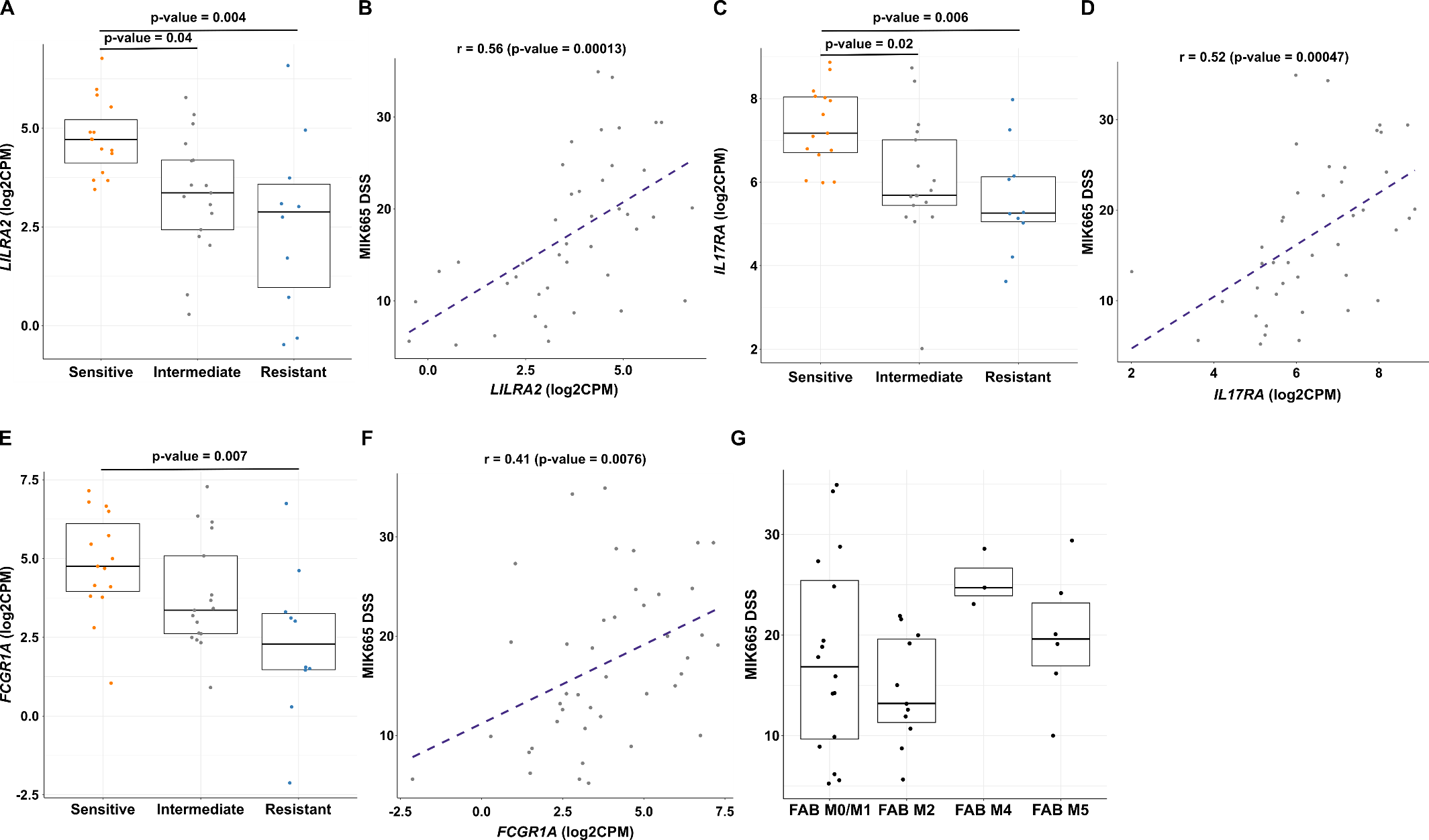


**Supplementary Figure 10. Differentiation-associated genes correlate with MIK665 sensitivity. A)** The median level of expression of *LILRA2* decreases significantly with MIK665 resistance (anova p-value = 0.0035) (MIK665-sensitive, *n* = 15; intermediate, *n* = 17; resistant, *n* = 10), **B)** and is significantly correlated with response to MIK665 (*n* = 42). **C)** The median level of expression of *IL17RA* decreases significantly with MIK665 resistance (anova p-value = 0.0036), **D)** and is significantly correlated with response to MIK665. **E)** The median level of expression of *FCGR1A* decreases significantly with MIK665 resistance (anova p-value = 0.0104), **F)** and is significantly correlated with response to MIK665. **G)** AML samples with a more differentiated FAB type (M4, *n* = 3; M5, *n* = 6) have higher median MIK665 DSS values than samples with a less differentiated FAB type (M0/M1, *n* = 16; M2, *n* = 11). Correlation tests were performed using the Spearman method and pairwise comparisons using the Tukey test. Log2CPM, log2 counts per million; DSS, drug sensitivity score; AML, acute myeloid leukemia; FAB, French-American-British.


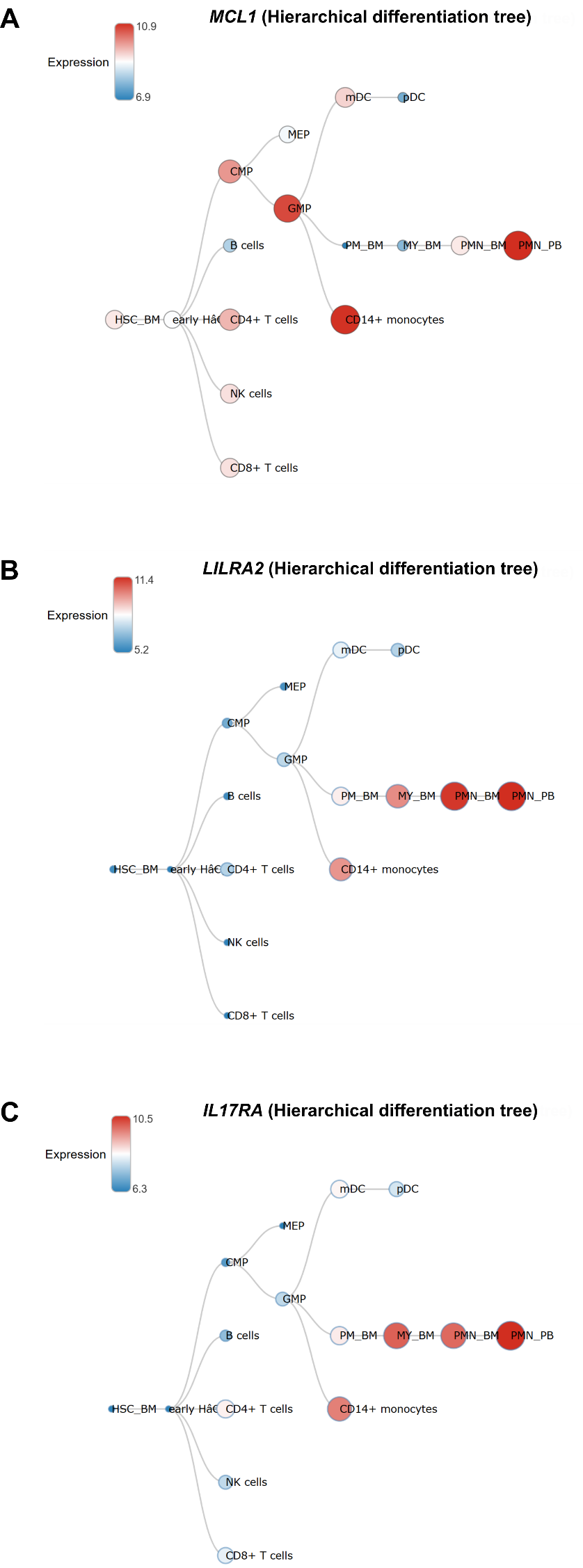


**Supplementary Figure 11. Hematopoietic trees demonstrating the increasing expression of A)** *MCL1*, **B)** *LILRA2* and **C)** *IL17RA* with differentiation in normal human hematopoiesis, notably towards cells of the monocytic and polymorphonuclear lineages (Source: HemaExplorer on BloodSpot). HSC_BM, Hematopoietic stem cells from bone marrow; early HPC_BM, Early hematopoietic progenitor cells from bone marrow; CMP, Common myeloid progenitor cell; GMP, Granulocyte monocyte progenitor cell; MEP, Megakaryocyte-erythroid progenitor cell; PM_BM, Promyelocyte from bone marrow; MY_BM, Myelocyte from bone marrow; PMN_BM, Polymorphonuclear cells from bone marrow; PMN_PB, Polymorphonuclear cells from peripheral blood; NK cells, natural killer cells; mDC, myeloid dendritic cells; pDC, plasma dendritic cell.


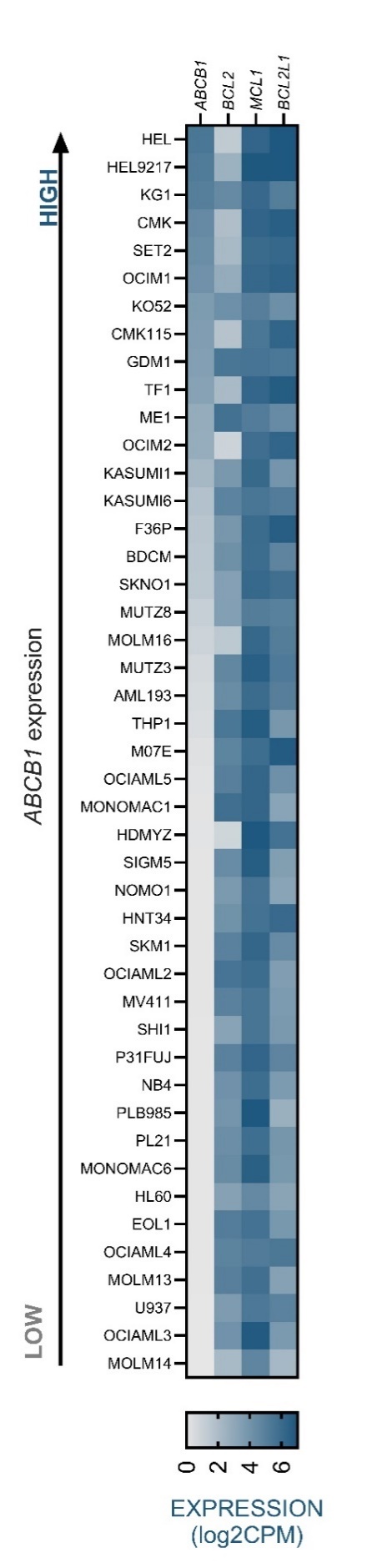


**Supplementary Figure 12.** **Heatmap showing *ABCB1*, *BCL2*, *MCL1* and *BCL2L1* expression in 45 AML cell lines.** The cell lines are sorted by *ABCB1* expression in log2CPM (Source: DepMap). Log2CPM, log2 counts per million; AML, acute myeloid leukemia.


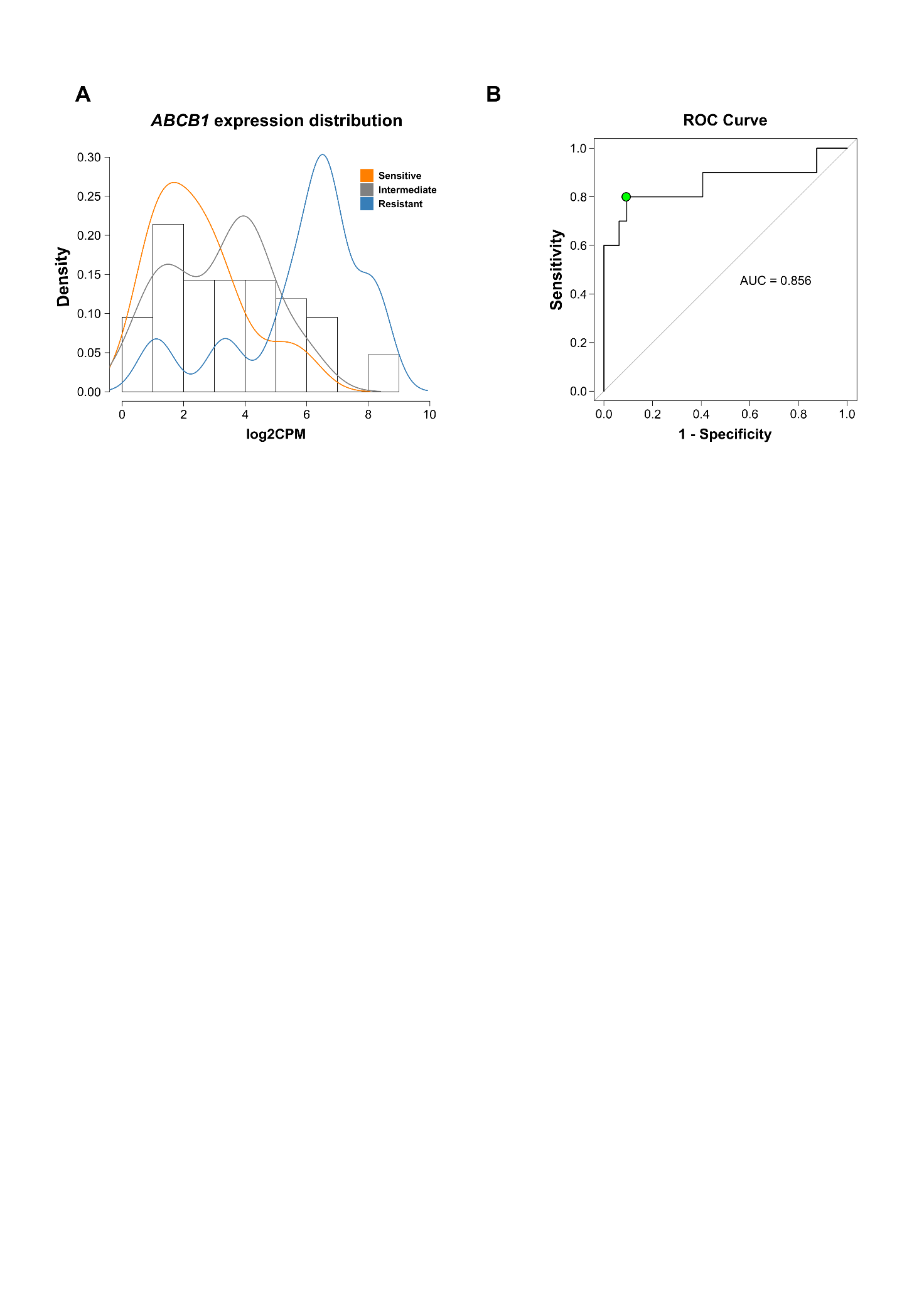


**Supplementary Figure 13. *ABCB1* gene expression can distinguish MIK665-resistant AML samples. A)** Density curves representing the distribution of *ABCB1* expression values across the MIK665 response groups. **B)** Receiver operator characteristic (ROC) curve of *ABCB1* expression as a predictor of MIK665 resistance. The optimal cutoff value for *ABCB1* was selected using the Youden index and is indicated by the green dot. log2CPM, log2 counts per million; AUC, area under the curve; AML, acute myeloid leukemia.

**
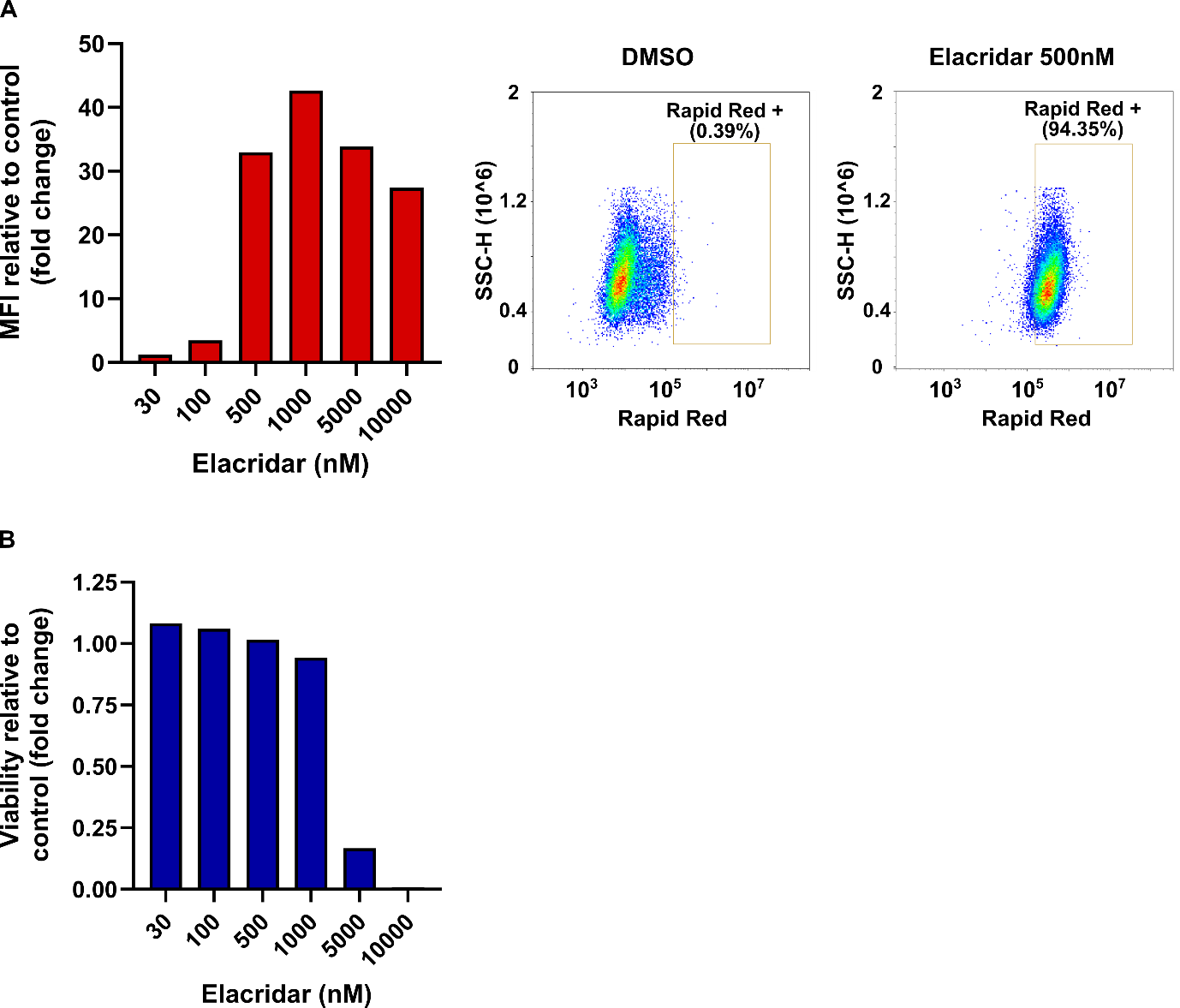
**

**Supplementary Figure 14. Flow cytometry-based drug efflux assay to assess the activity of increasing doses of elacridar in HEL cells. A)** Fold change in Rapid Red accumulation in HEL cells following treatment with increasing doses of elacridar for 24 h, as measured by mean fluorescence intensity (MFI), relative to DMSO (left) (*n* = 1). Representative plots of Rapid Red accumulation in HEL cells treated with DMSO or elacridar at 500nM (right). **B)** Fold change in viability of HEL cells relative to DMSO following treatment with increasing doses of elacridar for 24 h (*n* = 1). SSC-H, side scatter-height.


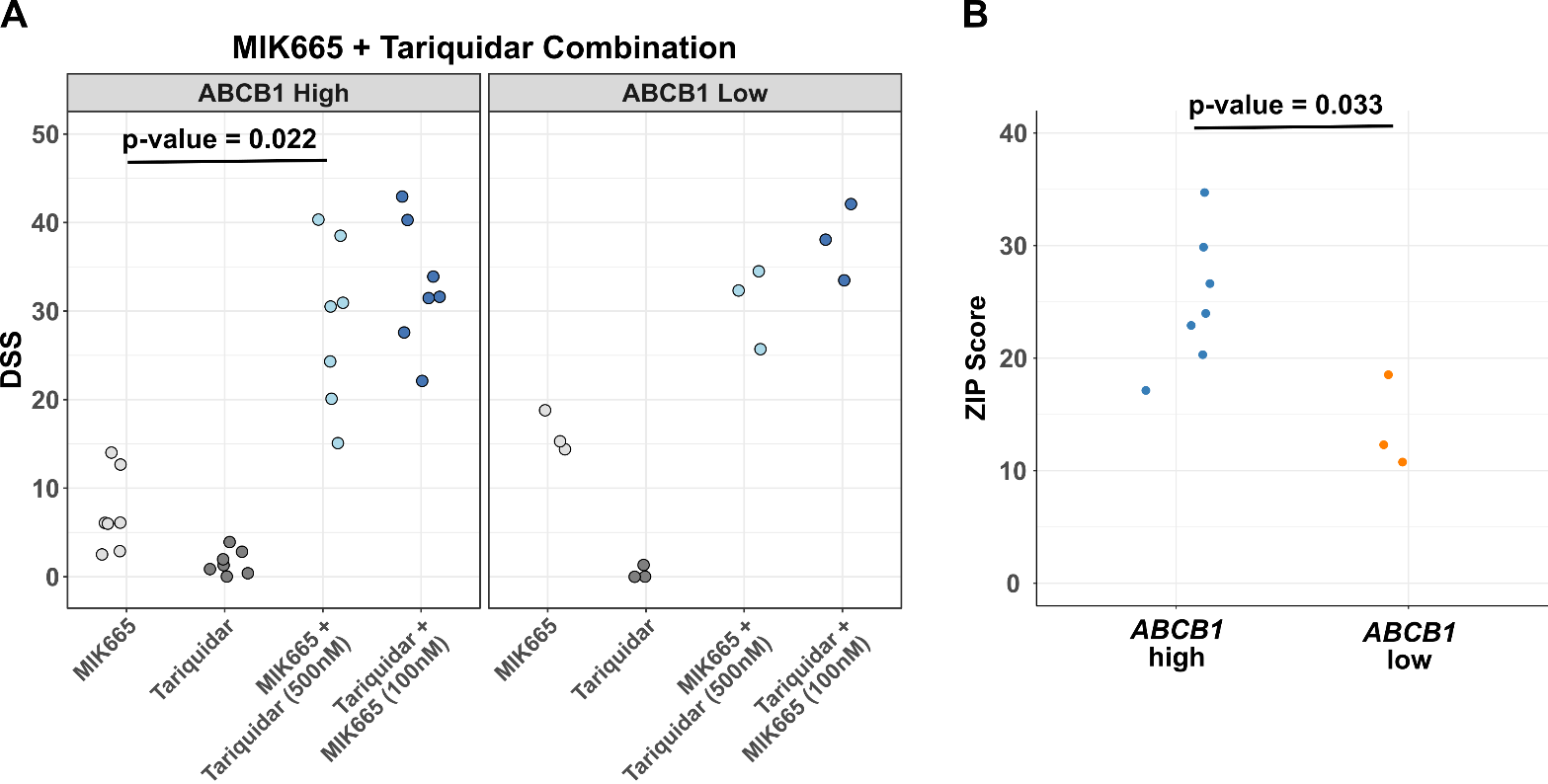


**Supplementary Figure 15.** **Results of the MIK665 and tariquidar combination testing in AML patient samples. A)** Dot plot showing the DSS of MIK665 and ABCB1 inhibitor tariquidar alone or in combination in AML samples with high (log2CPM > 5.22) (*n* = 7) or low (log2CPM < 5.22) (*n* = 3) *ABCB1* expression. Grey dots represent the DSS values of the single agents, whereas blue dots represent the DSS values of the combination (where one drug is increased along its concentration range while the other is fixed). Significance was evaluated using the paired sample t-test. **B)** Synergy dot plot showing ZIP scores for the MIK665 and tariquidar combination in *ABCB1* high or low samples. Responses were measured using flow cytometry following 48 h incubation with the drugs. DSS, drug sensitivity score; ZIP, zero interaction potency; AML, acute myeloid leukemia; log2CPM: log2 counts per million.

**
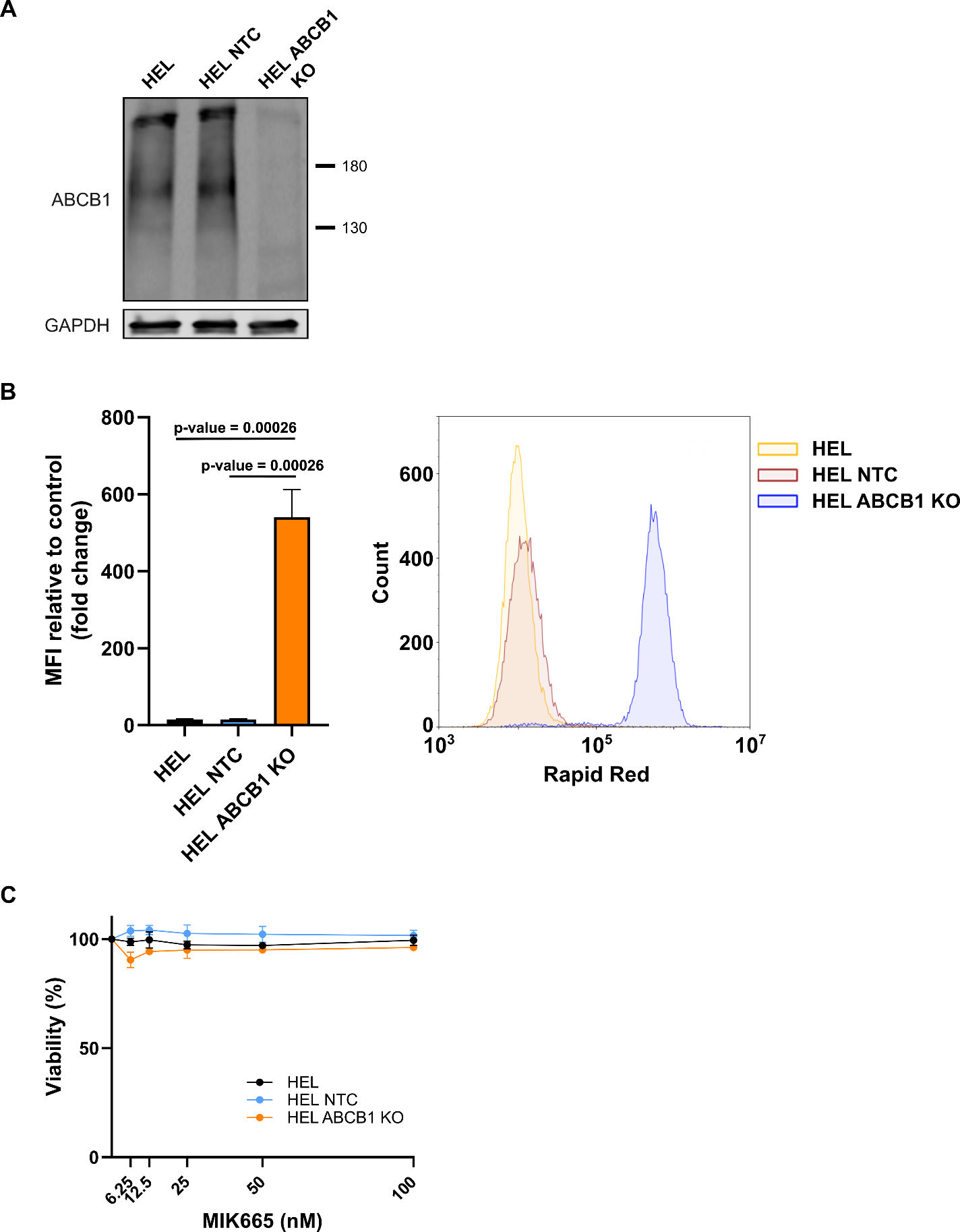
**

**Supplementary Figure 16. ABCB1 activity and MIK665 response in HEL ABCB1 knockout cells. A)** Western blot showing the protein expression level of ABCB1 in HEL ABCB1 knockout (HEL ABCB1 KO) cells, as compared to parental and non-targeting control (NTC) cells. **B)** Flow cytometry-based efflux assay showing the fold change in Rapid Red accumulation in HEL, HEL NTC, and HEL ABCB1 KO cells, as measured by mean fluorescence intensity (MFI), relative to DMSO, following a 24 h incubation (left) (*n* = 3). Representative density curve of Rapid Red accumulation in HEL, HEL NTC and HEL ABCB1 KO cells (right). **C)** Dose-response curves of HEL, HEL NTC and HEL ABCB1 KO cells following treatment with MIK665 for 48 h, measured using CellTiter-Glo (*n* = 3). Error bars represent the standard error of mean.


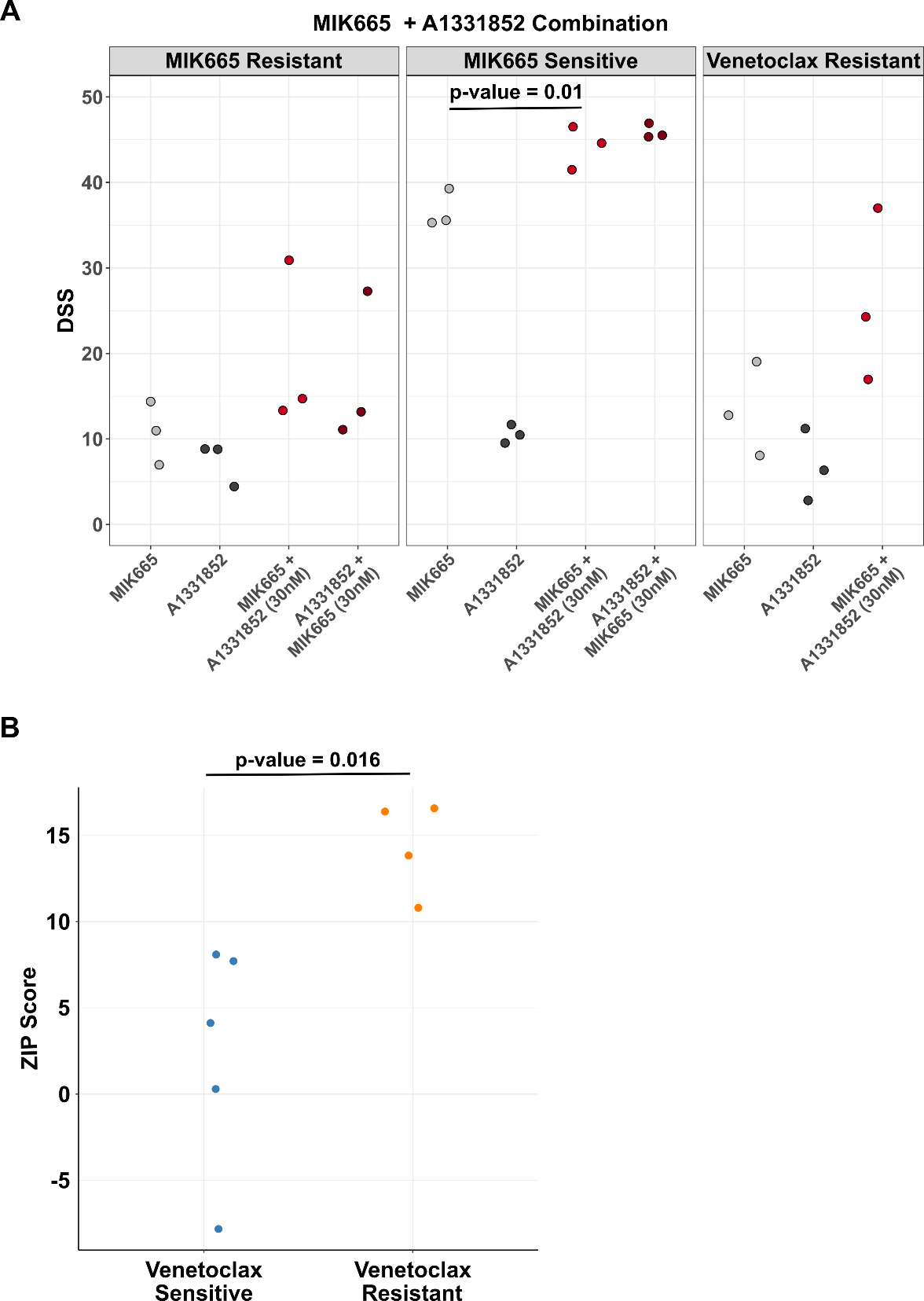


**Supplementary Figure 17. Results of the MIK665 and A1331852 combination testing in AML patient samples.** MIK665-resistant samples were selected as having a MIK665 DSS < 15 (*n* = 3), and MIK665-sensitive samples were selected as having a MIK665 DSS > 30 (*n* = 3). Venetoclax-resistant samples were selected as having a venetoclax DSS < 10 (*n* = 3). Grey dots represent the DSS values of the single agents, whereas red dots represent the DSS values of the combination (where one drug is increased along its concentration range while the other drug is fixed). Significance was evaluated using the paired sample t-test. **B)** Synergy dot plot showing ZIP scores for the MIK665 and A1331852 combination as grouped by venetoclax sensitivity (*n* = 5) and venetoclax resistance (*n* = 4) response. Significance was evaluated using the 2 sample t-test. DSS, drug sensitivity score; ZIP, zero interaction potency; AML acute myeloid leukemia.


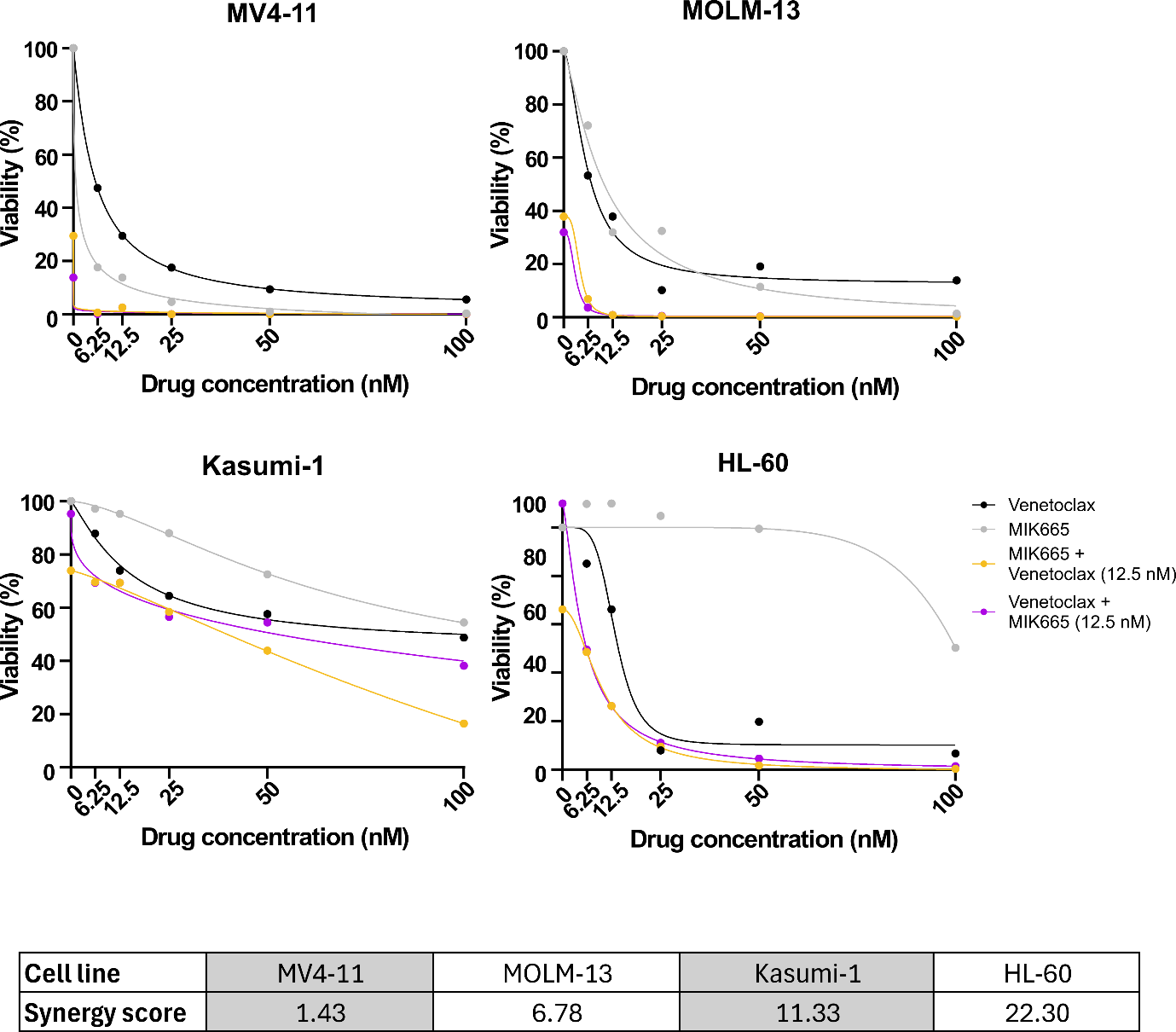


**Supplementary Figure 18. Results of the MIK665 and venetoclax combination testing in parental cell lines.** Dose-response curves of MIK665 and venetoclax alone and in combination in MV4-11, MOLM-13, Kasumi-1, and HL-60 cell lines, where one drug is increased along its concentration range while the other is fixed at 12.5 nM (*n* = 1). Responses are measured by CellTiter-Glo following 48 h incubation. ZIP synergy scores for the combination in each of the cell lines are reported in the table. ZIP, zero interaction potency.


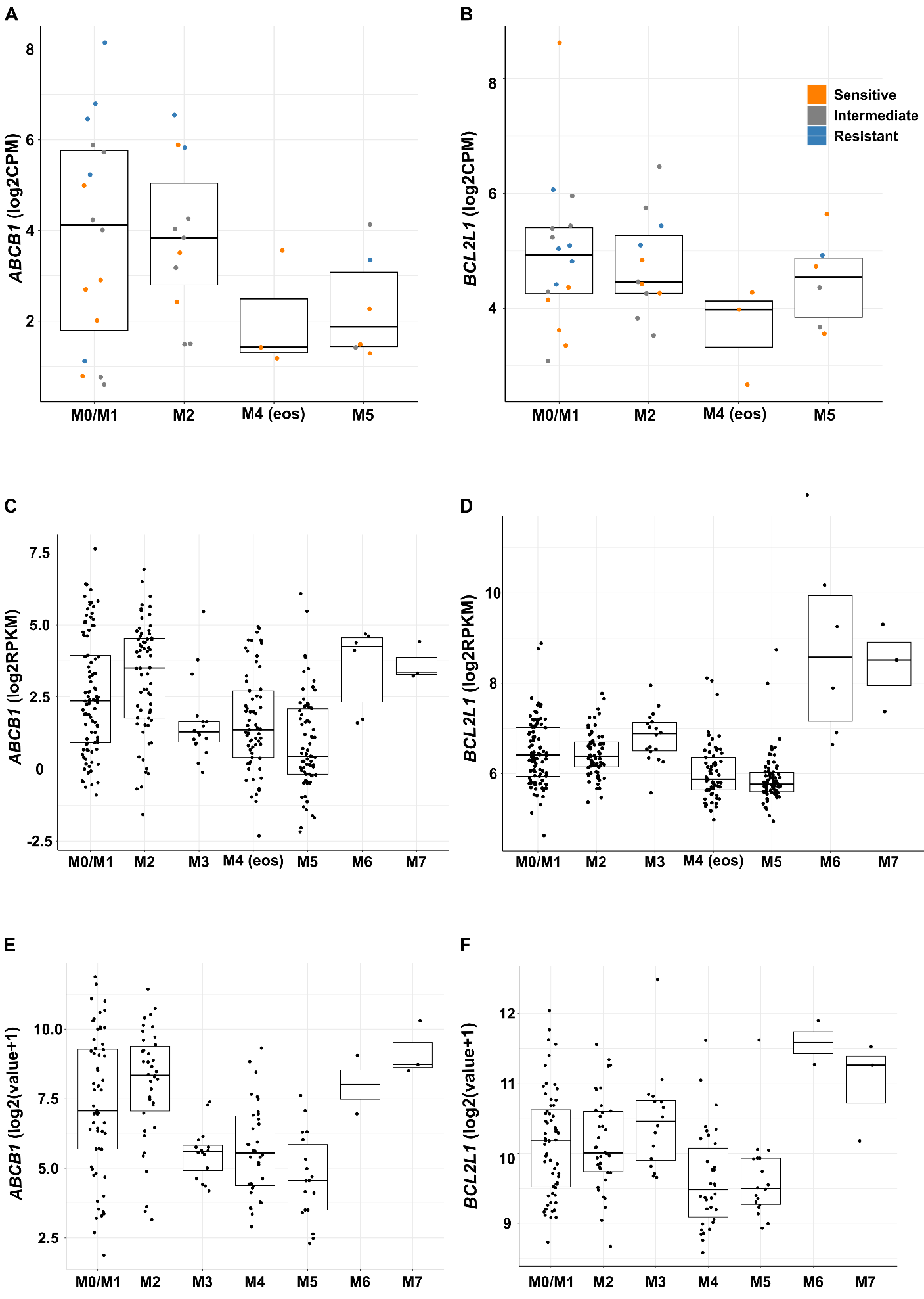


**Supplementary Figure 19. Expression of *ABCB1* and *BCL2L1* across FAB types of primary AML samples.** *ABCB1* and *BCL2L1* show higher median levels of expression in M0/M1 and M2 samples compared to M4 and M5 samples in **A-B)** **FIMM** (M0/M1, *n* = 16; M2, *n* = 11; M4 (eos), *n* = 3; M5, *n* = 6), **C-D)** **BEAT** (M0/M1, *n* = 94; M2, *n* = 66; M3, *n* = 18; M4 (eos), *n* = 65; M5, *n* = 74; M6, *n* = 6; M7, *n* = 3), and **E-F)** **TCGA** (M0/M1, *n* = 60; M2, *n* = 38; M3, *n* = 16; M4, *n* = 34; M5, *n* = 18; M6, *n* = 2; M7, *n* = 3) cohorts. The FIMM cohort samples are colored according to their response levels to MIK665. Significance was evaluated using the one-way anova test, followed by the Tukey test for pairwise comparisons. Log2CPM, log2 counts per million; log2RPKM, log2 reads per kilobase per million mapped reads; M4 (eos), bone marrow eosinophilia; AML, acute myeloid leukemia; FAB, French-American-British; FIMM, Institute for Molecular Medicine Finland; TCGA, The Cancer Genome Atlas.
